# Classification of osteoarthritic and healthy cartilage using deep learning with Raman spectra

**DOI:** 10.1038/s41598-024-66857-6

**Published:** 2024-07-10

**Authors:** Yong En Kok, Anna Crisford, Andrew Parkes, Seshasailam Venkateswaran, Richard Oreffo, Sumeet Mahajan, Michael Pound

**Affiliations:** 1 School of Computer Science, University of Nottingham, Nottingham, NG8 1BB UK; 2 Institute of Life Sciences and Department of Chemistry, University of Southampton, Southampton, SO17 1BJ UK; 3 Precision Healthcare University Research Institute, Queen Mary University of London, London, E1 1HH UK; 4 Bone and Joint Research Group, Centre for Human Development, Stem Cells and Regeneration, Institute of Developmental Sciences, University of Southampton, Southampton, SO16 6YD UK

**Keywords:** Raman spectra, Osteoarthritis, Deep learning, Convolutional neural network, Classification, Computational biology and bioinformatics, Computer science, Osteoarthritis

## Abstract

Raman spectroscopy is a rapid method for analysing the molecular composition of biological material. However, noise contamination in the spectral data necessitates careful pre-processing prior to analysis. Here we propose an end-to-end Convolutional Neural Network to automatically learn an optimal combination of pre-processing strategies, for the classification of Raman spectra of superficial and deep layers of cartilage harvested from 45 Osteoarthritis and 19 Osteoporosis (Healthy controls) patients. Using 6-fold cross-validation, the Multi-Convolutional Neural Network achieves comparable or improved classification accuracy against the best-performing Convolutional Neural Network applied to either the raw or pre-processed spectra. We utilised Integrated Gradients to identify the contributing features (Raman signatures) in the network decision process, showing they are biologically relevant. Using these features, we compared Artificial Neural Networks, Decision Trees and Support Vector Machines for the feature selection task. Results show that training on fewer than 3 and 300 features, respectively, for the disease classification and layer assignment task provide performance comparable to the best-performing CNN-based network applied to the full dataset. Our approach, incorporating multi-channel input and Integrated Gradients, can potentially facilitate the clinical translation of Raman spectroscopy-based diagnosis without the need for laborious manual pre-processing and feature selection.

## Introduction

Raman spectroscopy, a label-free and non-destructive technique, retrieves molecular vibrational information by utilising the inelastic scattering of photons from a sample upon monochromatic light radiation (typically with a laser). The 1-D Raman spectrum comprises characteristic peaks that correspond to the vibrational frequencies of molecular bonds, including functional groups and skeletal structures. This makes it possible to distinguish species of molecules^1^, thus providing a‘fingerprint’ of the chemical composition of the sample.

However, Raman signals can be severely affected by the analytical environment, as well as system and sample-dependent interferences such as cosmic rays, baseline shifts and overlapping bands. Numerous correction methods have been proposed for pre-processing the data to remove background noise or unwanted signals prior to analysis, offering better interpretation of the spectral data. Such pre-processing methods have been reviewed in detail and include baseline subtraction, cosmic ray removal, normalisation techniques, outlier rejection and spectral axis alignment^2^. Various studies have included a combination of these pre-processing steps as part of their standard pipeline for spectral analysis, and these generally outperform approaches based solely on raw data^3–5^.

Nonetheless, inappropriate selection of pre-processing strategies can remove or distort crucial spectral information, thus affecting analysis and resulting in misleading conclusions^6^. Since each dataset is different and will hold different artefacts, there is no ‘one size fits all’ approach, and the selection of pre-processing strategies is typically experience-dependent and is often optimised through a series of laborious trials.

Once data has been appropriately pre-processed, researchers traditionally employ conventional machine learning methods such as Principal Component Analysis (PCA), Linear Discriminant Analysis (LDA), Partial Least Squares (PLS), Cluster Analysis, k-Nearest Neighbor, Random Forest, Artificial Neural Network (ANN) and Support Vector Machine (SVM) for spectral classification and regression tasks^7,8^. While conventional machine learning methods utilise hand-engineered filters in pre-processing for better spectral distinction, a Convolutional Neural Network (CNN) can automatically learn this optimisation without human intervention. Previous studies^9–11^ showed that CNNs enable powerful learning from raw spectral features and achieved comparable or improved performance when compared to conventional machine learning methods trained on processed spectra. These approaches entirely avoid the pre-processing stage by designing an end-to-end deep learning method applied directly to raw data, whereas others^12,13^ implemented a CNN as a pre-processing approach prior to the subsequent quantitative or qualitative task.

To the best of our knowledge, the analysis of Raman spectra for the understanding and diagnosis of Musculoskeletal (MSK) diseases has so far been limited to conventional machine learning techniques on pre-processed spectra. Kumar et al.^14^ reported the use of PCA on the Raman spectra of human knee cartilage samples to classify the different stages of Osteoarthritis 43 (OA). Richardson et al.^15^ applied PCA-LDA on three pairs of ‘training’ Raman spectra i.e., one pair consisting of healthy and osteoarthritic human cartilage and the other two pairs consisting of biomolecules involved in modelling cartilage disease. They converted the spectral differences of these three pairs into multiple diagnostic metrics, which were then combined together to classify normal and OA human cartilage. Another study by Shaikh et al.^16^ implemented Partial Least Squares Discriminant Analysis to distinguish the types of cartilage injuries captured using Raman spectroscopy. There is one study^17^ that demonstrated the potential of deep neural network to study cartilage integrity in rabbits during OA using Near-infrared spectroscopy. However, no study has explored the application of deep learning techniques for the assessment of human MSK disorders based on Raman spectroscopy.

CNNs display complex structure and can learn complex non-linear functions. Though they are a powerful technique, analysis of the internal structure and learned weights is challenging, and, thus, CNNs are often regarded as a black box^18^. Recent studies^19–21^ have investigated approaches to interpret the representations of CNNs to help understand the regions of the spectra that have significant influence on the decision-making of the network. When applied to Raman spectra of diseased and healthy tissue, such approaches would enable the identification of important Raman bands/peaks that relate to specific biomarkers for disease diagnosis. Recognising these important Raman peaks is vital for spectral imaging applications, as the acquisition of spectral images using large number of wavenumbers can be costly and time-consuming. A practical approach to real-time acquisition of spectral imaging would be to capture a selected number of important wavenumbers that could be demonstrated to contribute the most information towards the disease characterisation.

In this work, we aim to provide a new method for Raman spectroscopy-based diagnostics by developing approaches that either avoid pre-processing entirely, or optimise pre-processing automatically via an end-to-end CNN with multi-channel input. The current studies demonstrate that such machine learning approaches offer strong performance, speed up analysis, can be done “blinded” and automated, reducing errors involved in common manual pre-processing steps. The approach also makes use of Integrated Gradients to identify key features used by the network, which correlate with the Raman signatures of biologically relevant molecules. By training on this restricted set of key features, we seek to demonstrate that the classification of OA and healthy cartilage is achievable, with performance comparable to models trained across all wavenumbers.

## Materials and methods

### Dataset

This retrospective study uses the dataset reported by Crisford et al.^22^ where the ethics approval, full research protocol, the dataset and its relevant details can be found. Femoral head specimens were collected from patients undergoing total hip arthroplasty at Southampton General Hospital (SGH) and Spire Southampton Hospital. All donors provided written informed consent prior to specimen collection. The study protocol received ethical approval from both the University of Southampton’s local Ethics and Research Governance Office (ERGO 71875) and the National Health Authority—North West—Greater Manchester East Research Ethics Committee (18/NW/0231). The study adhered to the ethical guidelines of the Helsinki Declaration. All work in this study was conducted in accordance with the relevant guidelines and regulations approved by the University of Southampton and the National Health Authority. All femoral heads were clinically evaluated and classified as either osteoarthritic (Mankin score 3 to 4) or non-osteoarthritic.

Briefly, osteoarthritic donors (n = 45, 24 female and 21 male) had no signs of osteoporosis or any other degenerative disease and the non-osteoarthritic donors (n = 19, 10 female and 9 male) had osteoporosis but no obvious detectable osteoarthritis or other cartilage degenerative disease and, hence were treated as‘healthy’ controls. Raman spectra of the superficial and deep layers were acquired for osteoarthritic and healthy cartilage harvested from these 45 OA and 19 Healthy patients. Each patient had 15–20 spectra recorded in the range of 614–1722 cm^−1^ (fingerprint region or named as Region A) and 2495–3264 cm^−1^ (CH_2_ stretching frequency region or named as Region B) from each of the layers. The original study^22^ used traditional machine learning methods (PCA and LDA) to analyse the dataset, including the use of manual pre-processing steps of Raman spectral signatures for OA diagnosis. The work achieved high classification accuracy for Region A but failed to achieve the same for Region B. This new study focuses on using deep neural networks to either remove the need for pre-processing entirely, or automatically identify and use the best pre-processing algorithm from among a common selection of approaches.

Table 1 shows the distribution of male, female, age and condition among the samples in the dataset.

We defined two datasets, one utilising the raw Raman data, and another using the pre-processing strategy of Crisford et al.^22^ as:

1. Raw Raman spectra
  - The data consists of 1015 data points of Raman spectral intensities vs wavenumbers for Region A and Region B.
2. Pre-processed Raman spectra
  - The data consists of 1011 data points of Raman spectral intensities vs wavenumbers for Region A and 1013 data points for Region B
  - Each spectrum was processed using the following pipeline as described in Crisford et al. work^22^:
    (i) 5th order polynomial to remove the fluorescent background
    (ii) Rubberband-like background subtraction to flatten ends of the spectrum
    (iii) Wavelet de-noising to smooth out spectra and eliminate high-frequency noise
    (iv) Vector Normalisation

Prior to evaluation, input spectra of all experiments in this study were standardised by subtracting the mean and dividing by the standard deviation of each spectral feature (wavenumber). This ensures that all spectral features have a mean of zero and a standard deviation of one.

### Proposed multichannel input

Prior works in OA diagnosis using Raman spectroscopy have often used a variety of pre-processing steps to improve the quality of the data before applying machine learning or other analysis. These steps are often necessary to extract the best performance of the downstream classification task, such as the classification of OA and Healthy controls. CNNs learn a highly non-linear mapping from the input space to the output, with increasing levels of abstraction added to each of the later networks. In theory, CNNs will automatically learn complex hierarchical features from the raw signal, transforming the input to improve separation between the different classes. However, common approaches to training, such as stochastic gradient descent, provide no guarantee that these complex functions will be learned, or provide optimal classification performance. We hypothesise that hand-engineered spectra correction algorithms are indispensable in guiding the learning process for robust analytical performance, particularly in the presence of a limited number of samples within a small dataset. We propose the design of a multi-channel input that comprises a mix of raw and pre-processed spectra, to effectively exploit the rich, complimentary, and sometimes redundant features of the differently pre-processed spectra.

The Raman data comprises over 1000 data points (Raman spectral intensity vs wavenumber) in Regions A and B, which we refer to here as input features. Our baseline models was trained on either raw or pre-processed input features representing a single channel input, thus for our baseline experiments the dimensionality of the input will be $1\times 1015$.

**Figure 1:**
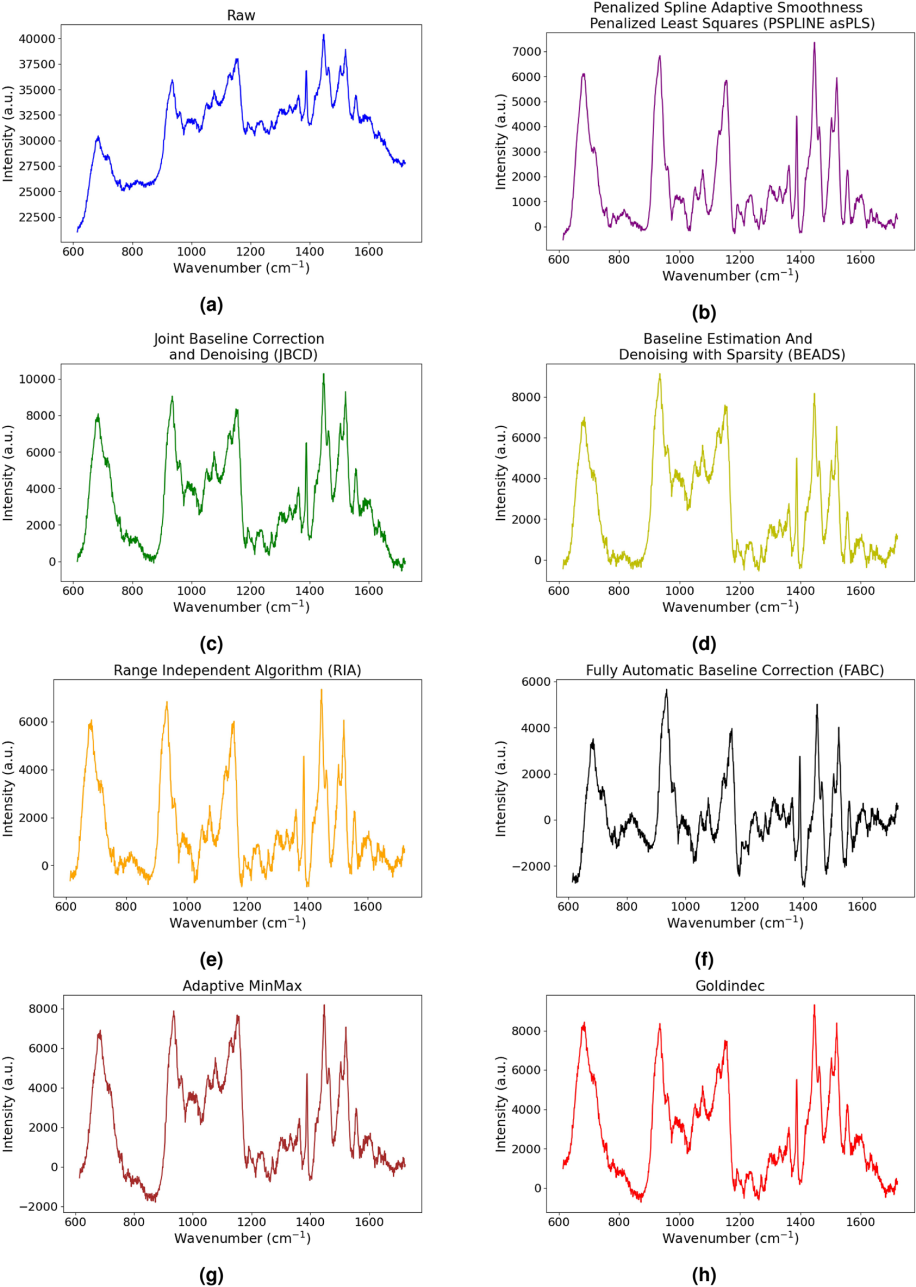
Sample of multichannel input. (**a**) is the raw spectra while (**b**–**h**) are the spectra processed using the correction method as specified in their title.

Our multi-channel approach comprises eight channels representing variations of the same spectra, each comprising 1015 wavenumbers, but with different pre-processing applied on each of the seven spectrum and one remaining in its raw form. The raw spectra is included as the first channel to ensure all information is preserved for the use by the network. Channels two to eight represent the same spectra after pre-processing using the seven most recent and robust categories of data correction algorithms (Table 2) in Pybaselines library (https://github.com/derb12/pybaselines). The Whittaker-smoothing and Spline based methods are similar, and hence is treated as a single category. A sample of our multichannel input prior to standardisation is depicted in Fig. 1.

### Network design

**Figure 2:**
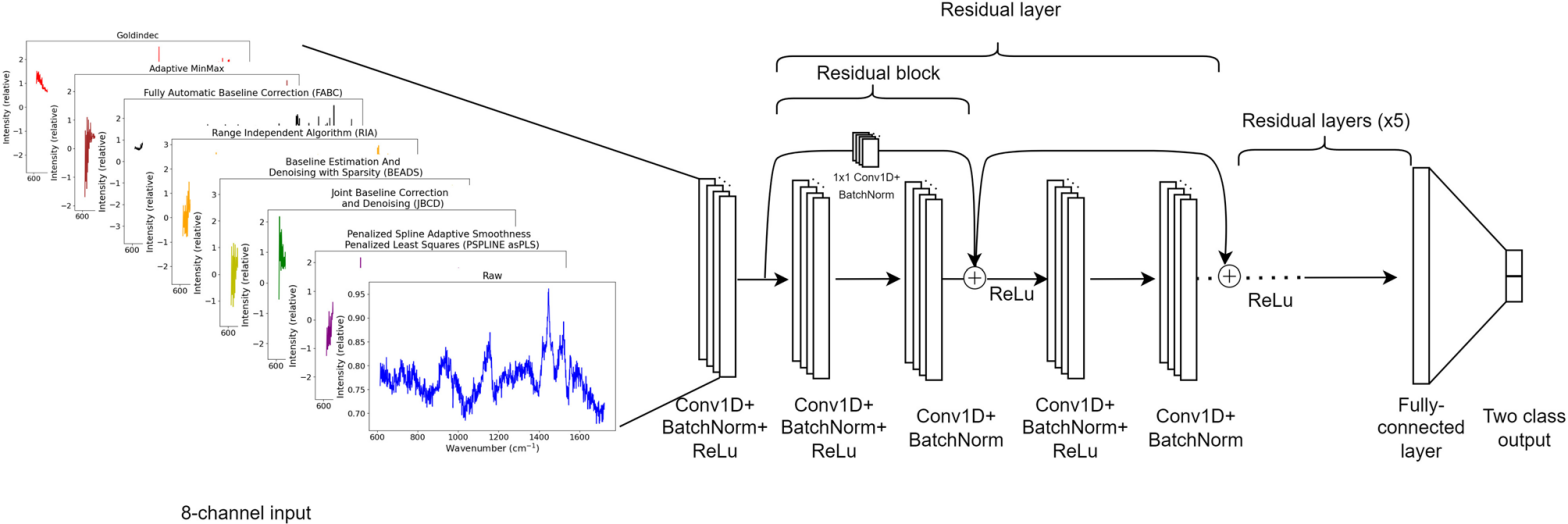
Our multi-channel CNN architecture takes in an 8-channel input, comprising of a mix of raw Raman spectra and those processed with different baseline correction methods. These are passed through a 1-dimensional Residual network for the classification of OA vs Healthy.

To account for the small sample size of our dataset (n = 406 to 2003 depending on the task), we implement a transfer learning strategy on all CNN-based networks used in this work. We employed a pretrained model from Ho et al.’s work^23^, which was originally trained on Raman spectra of pathogenic bacteria (n = 60,000). The 1-dimensional pretrained Residual network^24^ of Ho et al.’s work^23^ was adapted to include the multi-channel input by (i) modifying the initial convolutional layer to accept an 8-channel input (ii) initialising weights by duplicating the pre-trained weights from the original single-channel input (iii) replacing the final classification layer with a 2-class output. Figure 2 shows the Multi-CNN architecture used in this study.

For baseline comparison, we implement the same pretrained ResNet^23^, replacing only the final classification layer. The baseline network was trained on a single channel comprising either raw or handpicked pre-processed spectra.

### Feature selection

The input to our multi-channel CNN comprises 8 Raman spectra (one raw spectrum and seven with different pre-processing methods), each consisting of 1015 data points of wavenumbers vs intensity. Among these, some wavenumbers or (spectral features) will be unimportant to the eventual classification task, and others may be redundant or exhibit correlation between each of the eight inputs. Thus, Explainable Artificial Intelligence methods can aid understanding by visualising the input features that significantly influence the network’s predictions. This can facilitate the removal of irrelevant features for feature reduction purposes. We utilised Integrated Gradients to measure each wavenumber’s (spectral feature) contribution to the output decisions. Integrated Gradients^25^ have been previously shown to offer better interpretation robustness and reliability compared to other Explainable Artificial Intelligence methods^26^. The feature importance score is approximated using the integral of gradients of the model’s output with respect to the inputs along the path. In essence, the approach produce scores for each feature indicating their contribution to the eventual classification decision. We then reduced the number of training features based on their measured importance score to examine the impact of classification accuracy using ANN, (DT) and SVM classifiers. For our simple 3-layer ANN model, we utilised two hidden layers each containing 22 neurons. These were chosen following a grid search for the number of neurons in the hidden layers from values [10,12,14,16,18,22]. We applied the Rectified Linear Unit activation function on each of the hidden and output layers. This was then followed by a 20% dropout rate on the hidden layers. The (DT) and SVM classifiers were trained using default parameters from the scikit-learn^27^ library. We did not observe performance improvement by altering these parameters.

### Experimental design

We performed a stratified 6-fold cross-validation across all experiments, including the feature selection tasks. The dataset was split by patient subject to avoid samples from the same patient being used in both the training and testing sets. For each of the 6 splits, the training fold and the test fold comprised 80% and 20% of the data respectively. For the ANN and CNN models, we further split the “training” fold into 80/20 train and validation splits in order to better control the training process. Each model was trained for 100 epochs. The validation set was then used to determine the optimal number of epochs for training. Finally, we retrained the entire training set including the validation component and evaluated the model on the testing data, which was not used at any point during the training process. The training batch size for the CNN was set at 4, and the validation and test batch sizes were set at 1. Across all the experiments, we used cross entropy as the loss function and Adam optimizer with a learning rate of 0.0001 and betas (0.5, 0.999). To account for the imbalanced dataset of Healthy vs OA cartilage, the evaluation metric used is F1 score. The F1 score shown in all tables is the average F1 score of all test folds.

## Results and discussion

We first present results comparing the baseline CNN trained on either the raw or pre-processed dataset to our proposed Multi-CNN. This is then followed by the use of Integrated Gradients to rank the input features according to their contributions to the decision-making process summed over all 6 folds of the cross-validation process. Finally, using the ranked features, we produced subsets of the dataset comprising progressively smaller numbers of features from the full set of 1015 wavenumbers down to a single important feature. For each feature set, we evaluated the performance of ANN, DT, SVM on all classification tasks.

### Comparison between baseline CNN with proposed method

In the first experimental study we compared the baseline CNN applied to either the raw or pre-processed dataset against our proposed multi-channel method. The results of the layer-wise OA or Healthy cartilage classification and disease-wise superficial or deep layers assignment are detailed in Table 3. Regardless of approach, we notice a higher performance rate in Region A compared to Region B, supporting existing evidence that Region A has strong biochemical fingerprinting information^28^. The baseline raw approach generally shows lower performance compared to the other two methods, suggesting that the raw data may contain unwanted noise that can reduce classification accuracy. In the disease diagnosis tasks, the manually chosen pre-processing strategy often achieves better results than other methods, particularly in Region A. Whereas in the layer assignment tasks, our proposed multi-channel approach consistently outperforms other methods as well as exhibiting lower standard deviations, particularly in Region A. Overall, the baseline pre-processed approach shows strong performance across most tasks, likely due to its optimised selection of pre-processing strategies. Nevertheless, our flexible multi-channel approach offers improved or competitive performance, and does not require the laborious steps required to determine an optimal pre-processing strategy.

We performed additional experiments in which the dataset was split by gender (see results in Appendix A). Results across the different experiments are broadly similar to the all-gender experiment. The baseline pre-processed strategy demonstrates high F1-scores in most cases while our methodology provides improved or comparable results. However, we also note that some of the worse results by the baseline pre-processed approach in these smaller datasets may indicate that the manually chosen pre-processing method used here might not be the optimal strategy for all cases, as it may introduce bias.

We measure the computational cost of each approach to ensure a fair comparison. The Floating Point Operations Per Second (FLOPS) utilisation across all three methods exhibit negligible differences, with the baseline raw, pre-processed and our method having 0.401 GFLOPS, 0.399 GFLOPS and 0.403 GFLOPS respectively.

### Feature importance

**Figure 3:**
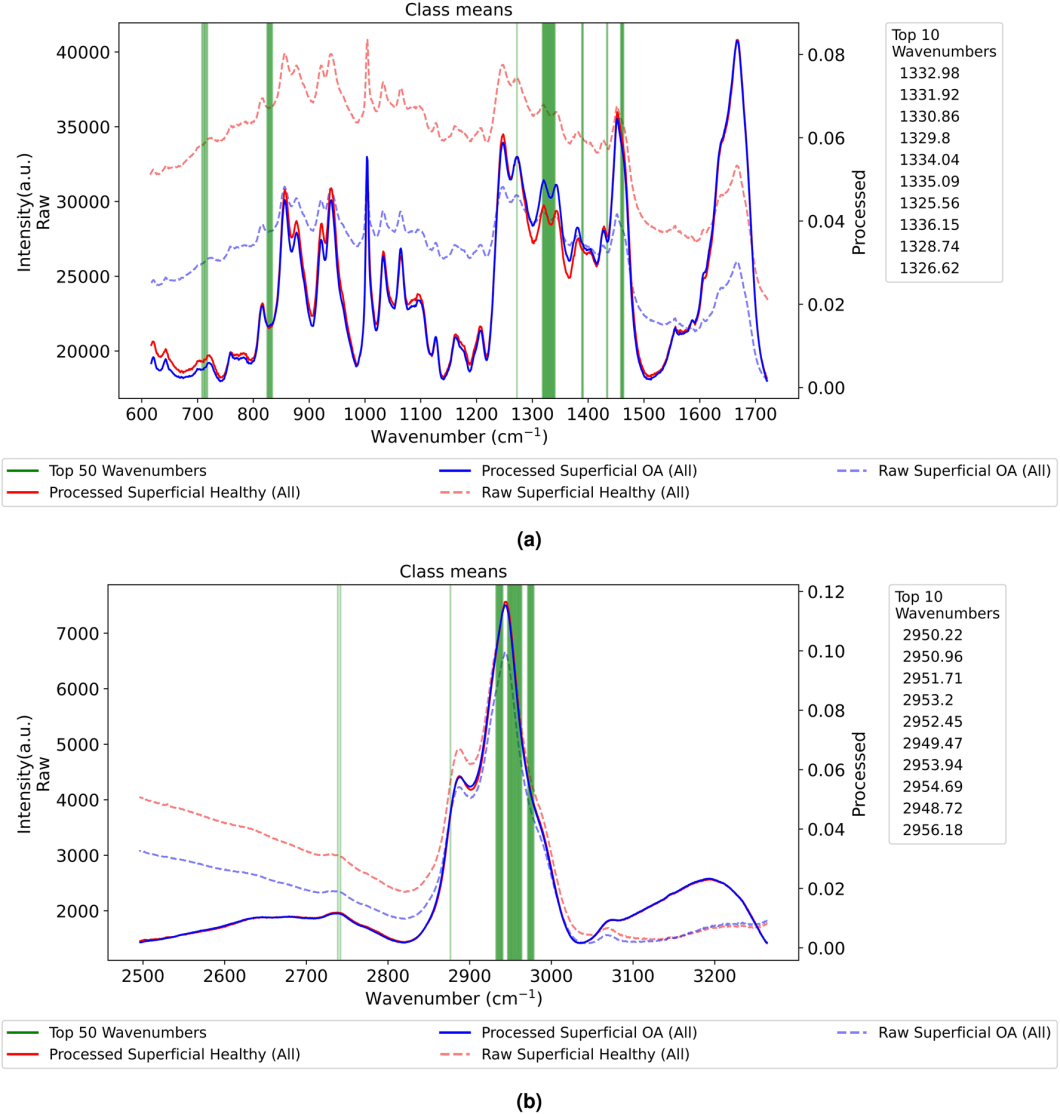
Sample cases of top 50 wavenumbers highlighted by the network in Superficial Healthy vs OA. (**a**) is recorded in the fingerprint Region A while (**b**) is in the CH_2_ stretching frequency Region B.

Integrated gradients were applied to the proposed multi-channel CNN across all classification tasks to rank the importance of input features, each representing a spectral feature corresponding to a wavenumber in the spectra. The top 50 wavenumbers identified as important are concentrated on specific regions of the spectrum, and correlate well to those known to be biologically relevant to human cartilage. In Fig. 3, we present two example outputs for Superficial Healthy vs OA cartilage at both A and B regions.

In the disease classification task at the Region A, the network highlights similar peaks in Deep Healthy vs OA and Superficial Healthy vs OA cartilage layers (see Fig.  3a and Fig. B1 at Appendix B). These common peaks include 1325–1335 cm^−1^ (Collagen wagging and twisting), 830 cm^−1^ (Proline and Hyrdroxyproline), ${\text{CH}}_{2}$ deformation (1437–1453 cm^−1^), and lipids and phospholipids (717–719 cm^−1^)^29–31^. The amino acids (Proline and Hyrdroxyproline) are the main components of type II collagen^32^, and all molecules mentioned have been identified as strong OA biomarkers in the literature ^30,33,34^. Our finding of the most prominent peak region (top 10 wavenumbers) 1325–1335 cm^−1^ (Collagen wagging and twisting) also aligns with the results by Crisford et al.^22^ who used a PCA-LDA method for classification on the same manually pre-processed dataset.

In Region B, most classification tasks commonly highlight several regions between 2885–3000 cm^−1^ that suggest CH, CH_2_ and CH_3_ stretch^35^ (see Fig. 3b and Fig. B12 to B22 at Appendix B) and likely refer to lipid and protein contents in the articular cartilage ^29,36,37^.

Additional plots showing the top 50 wavenumbers highlighted by the network for other experiments, including layer assignment and disease diagnosis tasks with gender specificity, are provided in Appendix B. In general, these additional experiments reveal prominent peaks with high similarity to the results of Crisford et al.^22^ and strongly correlate with established biomarkers identified in the literature.

### Feature selection

We compared ANN, DT and SVM for feature selection using a stratified 6-fold cross-validation. We discuss the results here, and present detailed results in Appendix C. Based on our analysis, we observe a higher performance rate by ANN in most classification tasks. Whereas DT and SVM demonstrate a more uniform performance across training runs and little to no performance drop even as we reduce the number of features.

In all disease classification tasks, we notice that reducing the number of features does not always affect the classification performance negatively and the overall performance is stable across the different algorithms. Using only 1–3 features, ANN and SVM achieve performance comparable to the best-performing CNN-based network applied to the full dataset in most classification tasks. Additionally, in some cases involving disease classification in Region B, ANN or SVM even outperform the best CNN-based network when using the reduced feature set.

In the layer assignment tasks, ANN often show notably better performance than SVM and DT, although its performance is slightly unstable. This instability can be attributed to the network’s sensitivity to outliers, which is exacerbated by its development on a small sample size. Experimental results show that the performance rate of these algorithms increases rapidly as we increase the number of features up to a certain point. The best-performing algorithm, ANN, in this case, takes around 50 and 300 feature sets respectively for the all-gender and gender-specific classification tasks to achieve comparable accuracy to the CNN-based networks. This can be explained by the fact that ANN might need more features to learn the complex relationships necessary for accurate classification on the smaller dataset of the gender-specific tasks.

**Figure 4:**
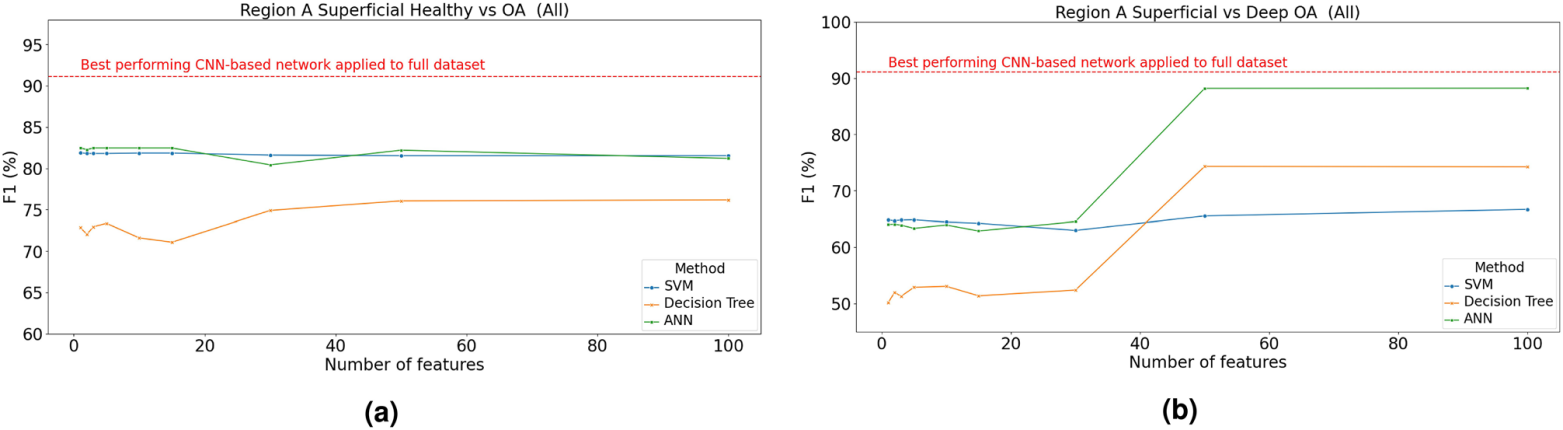
Sample cases of feature selection using SVM, Decision Tree or ANN at Region A: (**a**) Superficial Healthy vs OA, (**b**) Superficial vs Deep OA.

Figure 4 shows the two example outputs for the experiments using 1 to 100 feature sets in the disease classification and layer assignment tasks using data in the fingerprint region (Region A). Detailed plots demonstrating the feature selection experiments for all classification tasks may be found in Appendix C.

In general, our experiments show that by using just a few features, basic machine learning algorithms such as ANN can attain accuracy comparable to the best-performing CNN-based network applied to the full dataset. This implies that the feature selection method (i.e. calculation of feature importance score by applying Integrated Gradients on proposed Multi-CNN) can be a useful to in eliminating redundant or noisy features, and potentially enhancing classification accuracy. Additionally, this further confirms that the features deemed to be important by our proposed method were relevant for the decision-making in these classification tasks. Notably, in a clinical setting, this approach could translate to significant time and cost savings by enabling clinicians to utilise a reduced set of essential wavenumbers during measurement and analysis procedures.

## Conclusion

The current studies demonstrate a fully automatic CNN solution applied to Raman spectroscopy that provides improved OA classification against healthy controls and assignment to superficial or deep layers based on disease or healthy controls. The approach speeds up analysis and reduces potential error involved in manual selection of pre-processing steps, which traditionally demand weeks of human labor and cannot be executed in a completely “blinded” manner. Results show that the proposed methodology achieved similar or better performance compared to a baseline CNN trained on either the raw or fixed pre-processed spectra. The proposed method removes the need to handpick “optimal” pre-processing strategies by enabling the network to automatically learn a good combination of pre-processed features, while also incorporating the raw spectra to avoid any loss of critical information. We next utilised Integrated gradients to show that our proposed Multi-CNN makes predictions based on biologically relevant spectral features corresponding to specific wavenumbers in the Raman spectra. By selecting on a small subset of those that were ranked as most important, we were able to train additional classifiers to a very similar accuracy to those trained on the full feature input. Maintaining high performance with only a small number of spectral features suggests that our approach may be suitable for Raman-based imaging, in which each additional spectral feature incurs a cost in terms of time required to capture imaging data. Future work will explore the wider application of the proposed Multi-CNN on spectroscopy data from other domains, where we anticipate that multi-channel input will improve accuracy and robustness. We will also explore alternative ways to combine multi-channel inputs, such as attention mechanisms, to work towards performance that never falls below either raw or static pre-processing. The improved and rapid classification using our approach could potentially enable the translation of Raman spectroscopic approaches to the clinic and help practitioners improve outcomes for patients.

**Table 1:** Population sample data of Osteoarthritis and Healthy patients.

	Male	Female
Osteoarthritis		
Count, no.	21	24
Age, mean±SD (range), years	69.62±10.33 (49-83)	68.92±13.21 (40-87)
Healthy		
Count, no.	9	10
Age, mean±SD (range), years	72.00±14.33 (47-88)	71.30±17.82 (40-88)

**Table 2:** Baseline correction algorithms selected for the construction of the multichannel input from the raw spectra.

Category	Algorithm	Description
Whittaker-smoothing and Spline	Penalized Spline Adaptive Smoothness Penalized Least Squares(PSPLINE asPLS)^38,39^	Penalized spline version of asPLS to balance the fidelity and smoothness of the fitted baseline with an adaptive smoothing parameter based on the peak and non-peak regions.
Morphological	Joint Baseline Correction and Denoising (JBCD)^40^	Uses mathematical morphological operations along with regularised least-squares fitting for the removal of baseline distortion and the estimation of a smooth spectrum.
Smoothing	Range Independent Algorithm (RIA)^41^	A range independent background-subtraction algorithm that iteratively applies a Savitzky-Golay smoothing method (moving point average) on the spectra. This gradually eliminates the high frequency peaks, allowing the broad underlying baseline to be subtracted from the raw spectrum, thus yielding the true signal.
Classification	Fully Automatic Baseline Correction (FABC)^42^	It relies on the automatic recognition of signal-free regions to implement a Continuous Wavelet transform algorithm combined with the Whittaker smoothing algorithm for baseline modelling. It can automatically flatten the spectra with significant baseline distortion and is robust against spectra with low signal-to-noise ratios and varying widths.
Optimizer	Adaptive MinMax^43^	It selects the subtraction technique based on the fluorescence-to-signal ratio, effectively reducing RMS error while dealing with different fluorescence-to-signal ratio.
Polynomial	Goldindec^44^	An iterative algorithm that generates parameters automatically from raw data to fit the baseline without being affected by large peaks, peak number or wavenumber.
Miscellaneous	Baseline Estimation And Denoising with Sparsity (BEADS)^45,46^	It performs baseline correction and noise reduction by modelling the baseline as low-pass signal and the noise as high-pass contribution, while the peaks are considered as sparse with sparse derivatives.

**Table 3:** F1 Comparison of baseline CNN on raw and pre-procesed dataset with the proposed method using a 6-fold cross-validation in Region A and B on disease and layer classification tasks.

Region	A			B		
Methodology	Raw	Pre-processed	Ours	Raw	Pre-processed	Ours
Disease diagnosis						
Superficial Healthy vs OA	83.43 ± 3.78	**85.98** ±** 5.02**	84.05 ± 4.05	81.01 ± 3.42	**81.64** ± **2.63**	81.12 ± 3.08
Deep Healthy vs OA	84.12 ± 3.37	**89.34** ±** 4.01**	82.98 ± 3.17	**83.45** ±** 3.60**	81.24 ± 5.06	80.38 ± 3.80
Layer assignments						
Superficial vs Deep Healthy	92.24 ± 6.04	93.49 ± 5.39	**94.12 **± **5.36**	88.10 ± 5.43	88.56 ± 5.17	**88.95 **± **7.13**
Superficial vs Deep OA	89.25 ± 6.52	90.03 ± 6.42	**90.59** ± **5.66**	86.97 ± 5.85	**89.39 **±** 5.16**	86.84 ± 5.71

### Supplementary Information

Supplementary Information.

## Data Availability

The code and dataset are available at https://github.com/janetkok/Raman_spectra_classification_of_OP_and_OA.
