# Supplementary material for: Classification of osteoarthritic and healthy cartilage using deep learning with Raman spectra
Source: Sci Rep. 2024 Jul 10;14:15902. doi: 10.1038/s41598-024-66857-6 (PMC11237049; doi:10.1038/s41598-024-66857-6)
Supplement: Supplementary file 1 — Supplementary Information. [file 41598_2024_66857_MOESM1_ESM.pdf]

Appendix A

Region	A			B		
**Methodology**	Raw	Pre-processed	Ours	Raw	Pre-processed	Ours
**Disease diagnosis:**						
**Superficial Healthy vs OA**						
Men	**80.51±5.12**	78.97±8.61	77.66±7.31	75.70±7.44	76.47±11.87	**78.20±9.41**
Women	85.73±5.84	**91.54±6.56**	87.56±3.98	81.46±6.38	**86.99±6.48**	82.31±4.60
**Deep Healthy vs OA**						
Men	82.20±4.74	83.84±4.54	**84.92±5.99**	81.31±7.84	83.93±5.28	**84.68±6.06**
Women	85.90±6.34	**90.63±5.75**	88.85±6.04	**83.25±8.42**	81.53±6.04	82.84±6.90
**Layer Assignments:**						
**Superficial vs Deep Healthy**						
Men	88.65±8.82	**95.66±5.08**	90.41±7.95	85.48±10.95	**93.08±4.00**	89.02±9.28
Women	83.00±16.22	**90.25±8.83**	86.86±14.26	67.06±12.99	**86.28±10.71**	72.79±11.66
**Superficial vs Deep OA**						
Men	93.88±3.18	96.58±2.42	**96.64±2.72**	**93.01±3.99**	91.98±5.21	91.33±5.31
Women	77.96±6.40	**85.27±6.23**	84.36±6.58	78.51±7.06	79.33±8.60	**79.71±9.63**

Table A1. F1 Comparison of baseline CNN on raw and pre-procesed dataset with the proposed method using a 6-fold cross-validation in Region A and B on disease and layer classification tasks with gender specificity.

Appendix B

Top 50 wavenumbers (features) highlighted by Multi-CNN in different classification tasks.

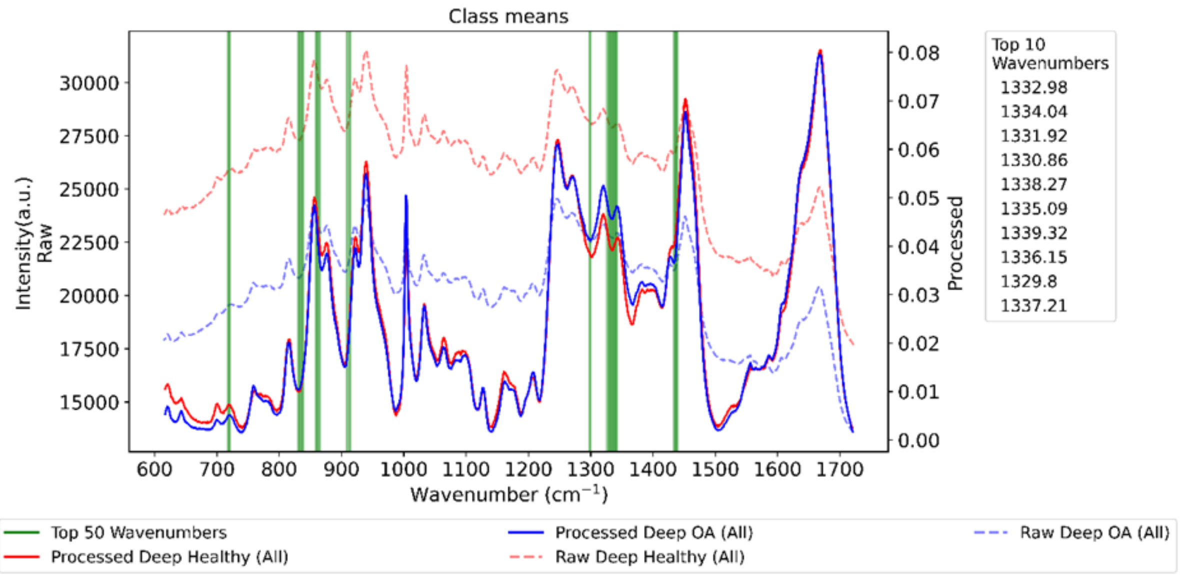

Figure B1 Top 50 wavenumbers highlighted by Multi-CNN at Region A: Deep Healthy vs OA (All)

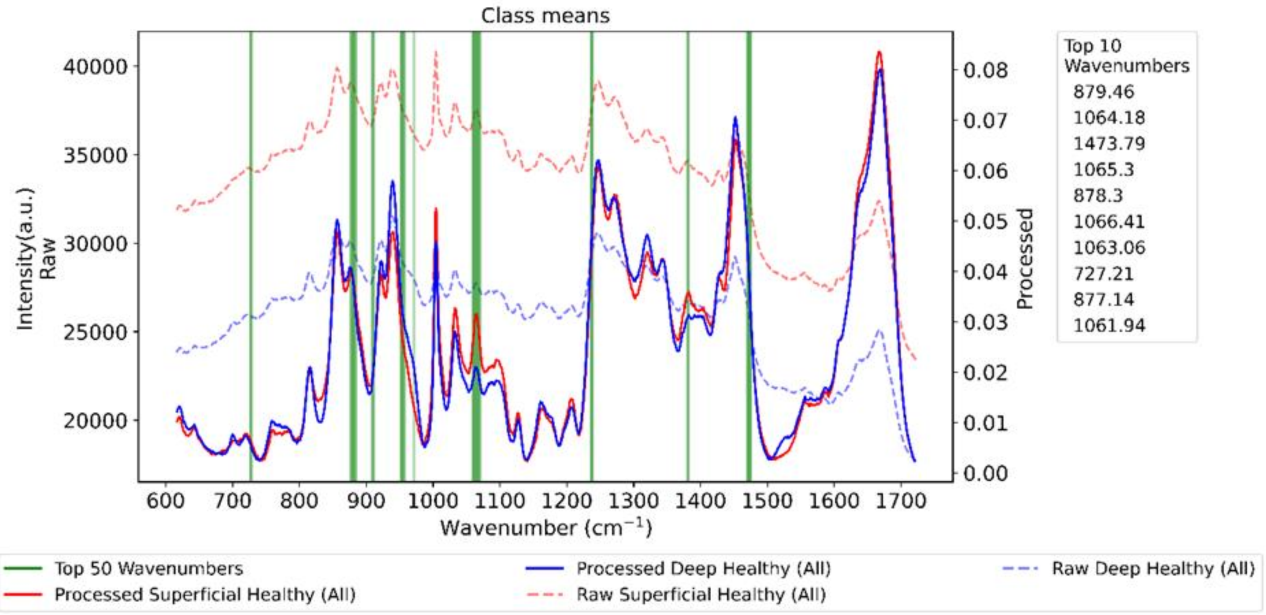

Figure B2 Top 50 wavenumbers highlighted by Multi-CNN at Region A: Superficial vs Deep Healthy (All)

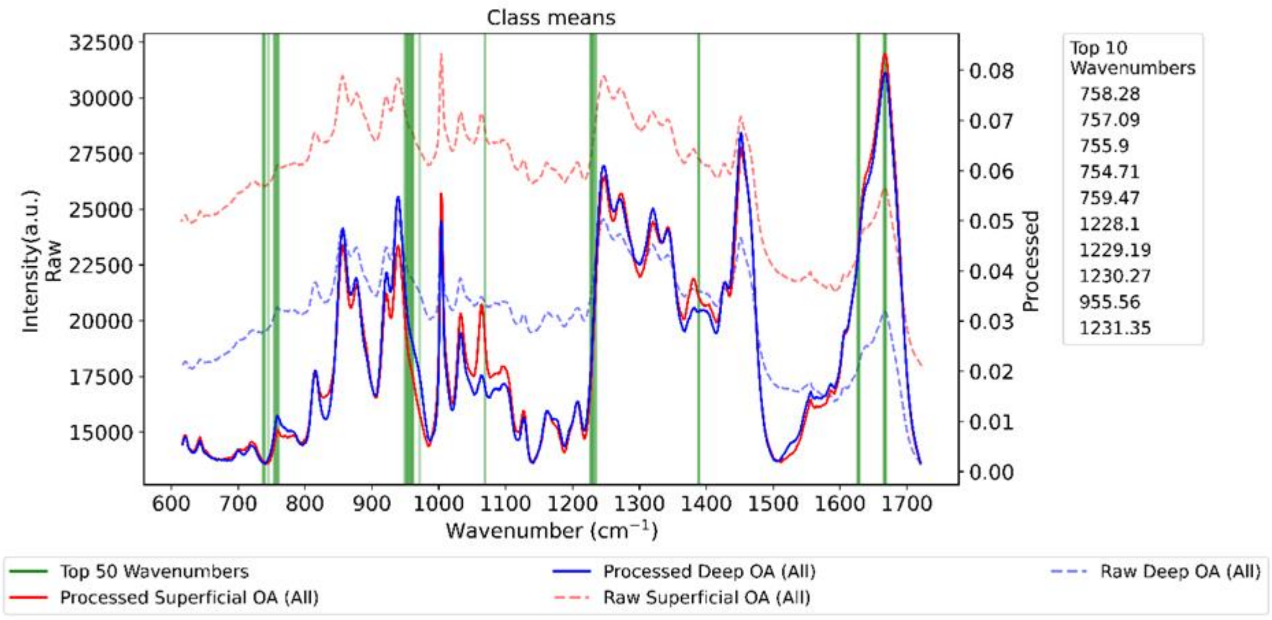

Figure B3 Top 50 wavenumbers highlighted by Multi-CNN at Region A: Superficial vs Deep OA (All)

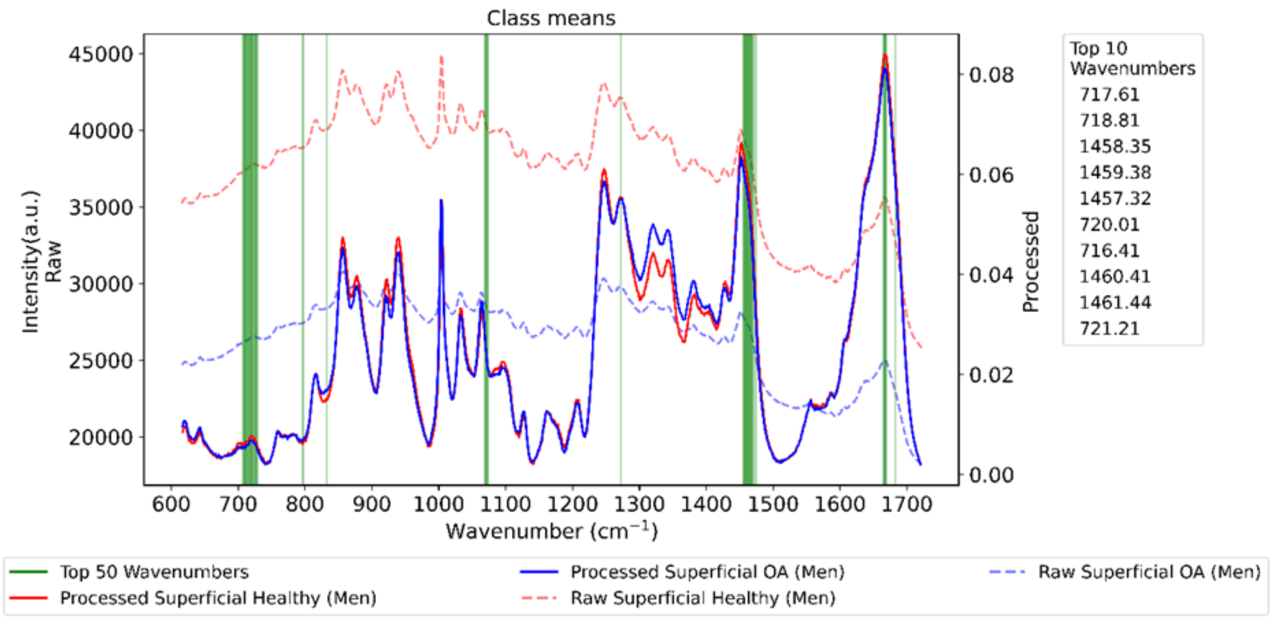

Figure B4 Top 50 wavenumbers highlighted by Multi-CNN at Region A: Superficial Healthy vs OA (Men)

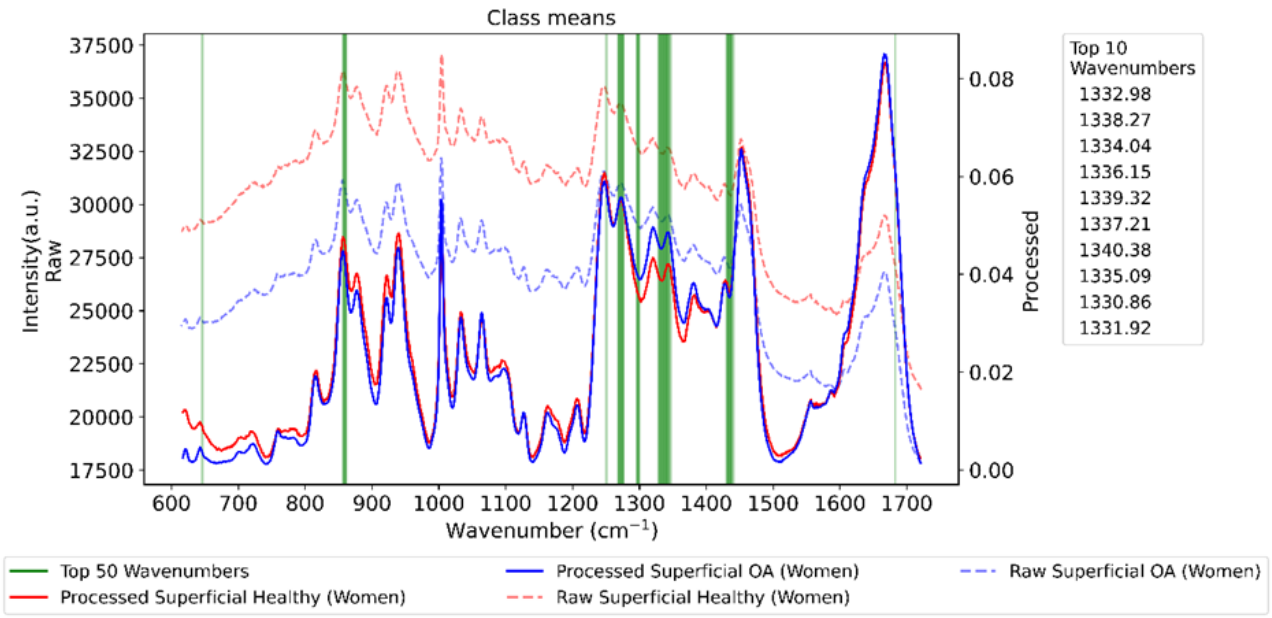

Figure B5 Top 50 wavenumbers highlighted by Multi-CNN at Region A: Superficial Healthy vs OA (Women)

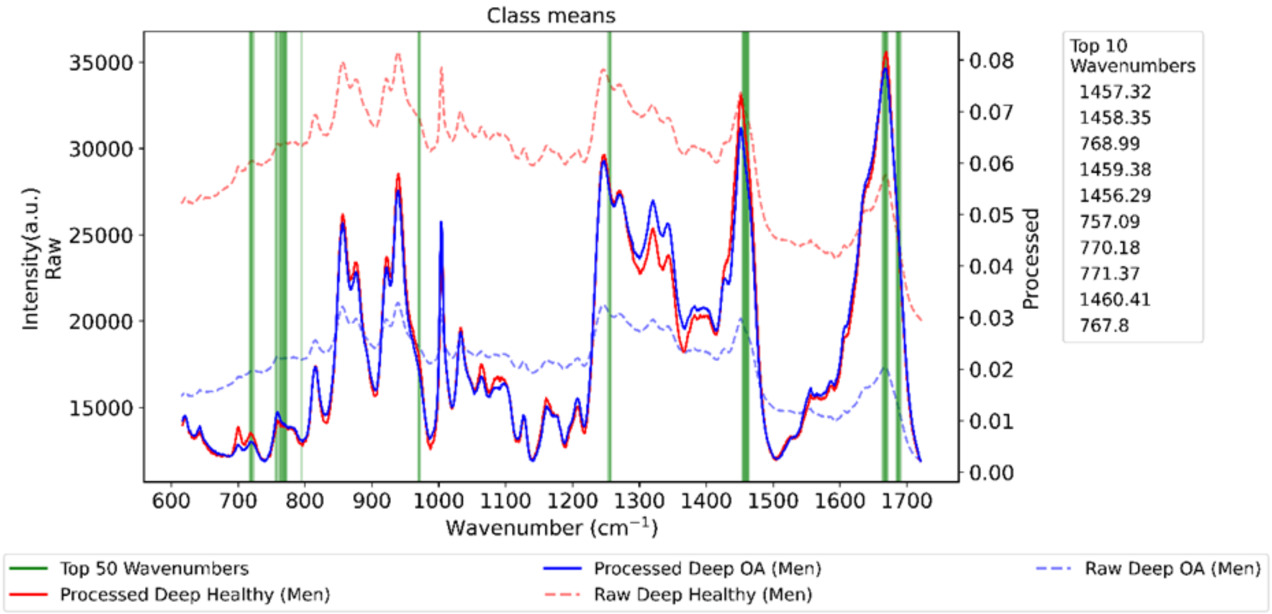

Figure B6 Top 50 wavenumbers highlighted by Multi-CNN at Region A: Deep Healthy vs OA (Men)

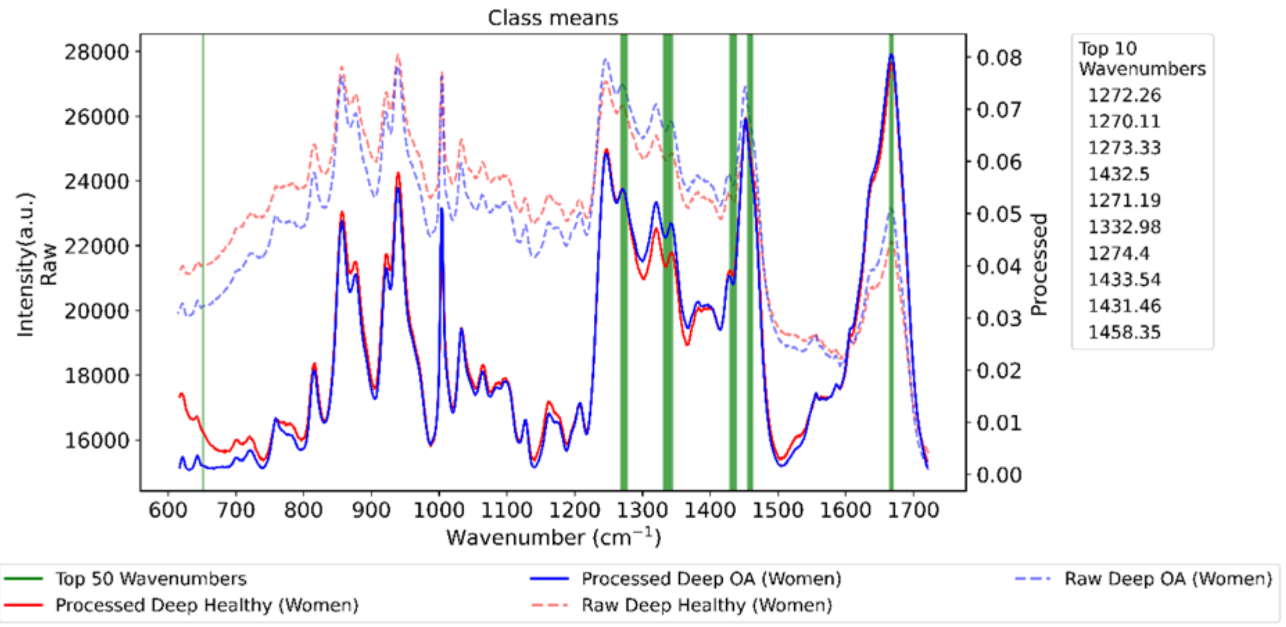

Figure B7 Top 50 wavenumbers highlighted by Multi-CNN at Region A: Deep Healthy vs OA (Women)

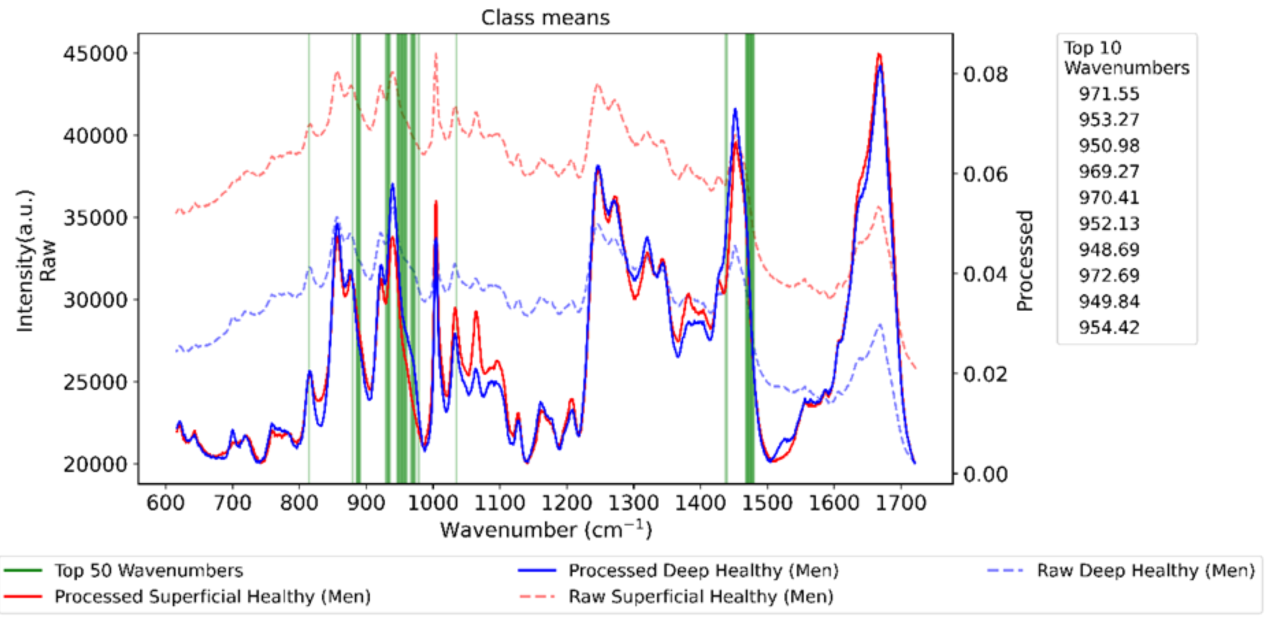

Figure B8 Top 50 wavenumbers highlighted by Multi-CNN at Region A: Superficial vs Deep Healthy (Men)

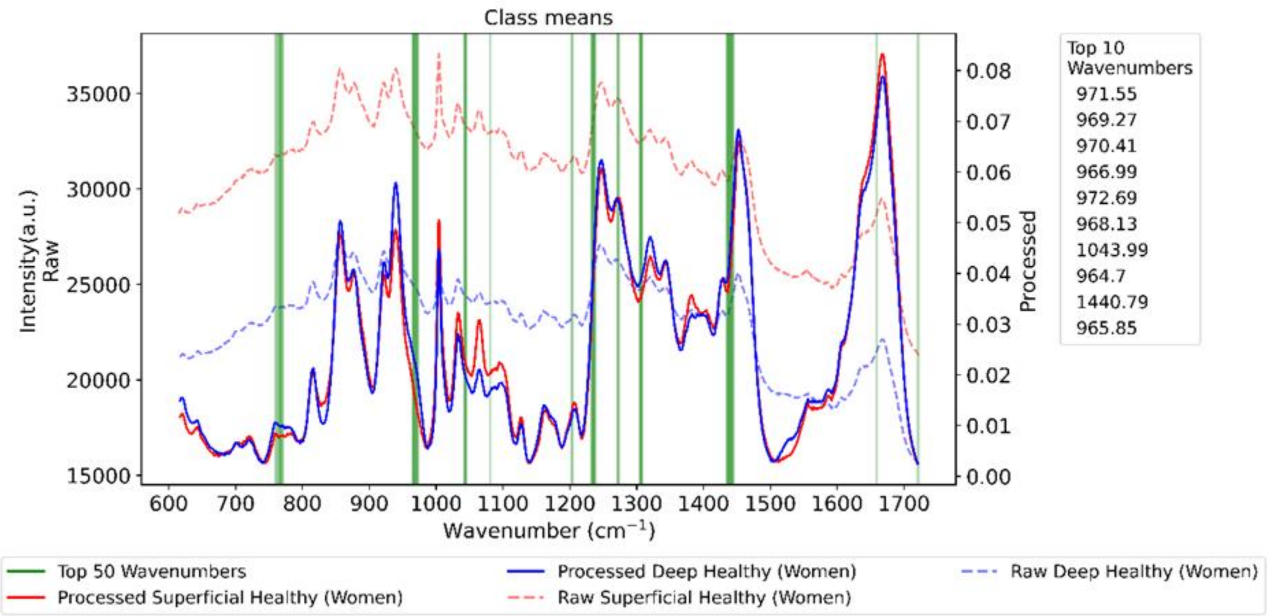

Figure B9 Top 50 wavenumbers highlighted by Multi-CNN at Region A: Superficial vs Deep Healthy (Women)

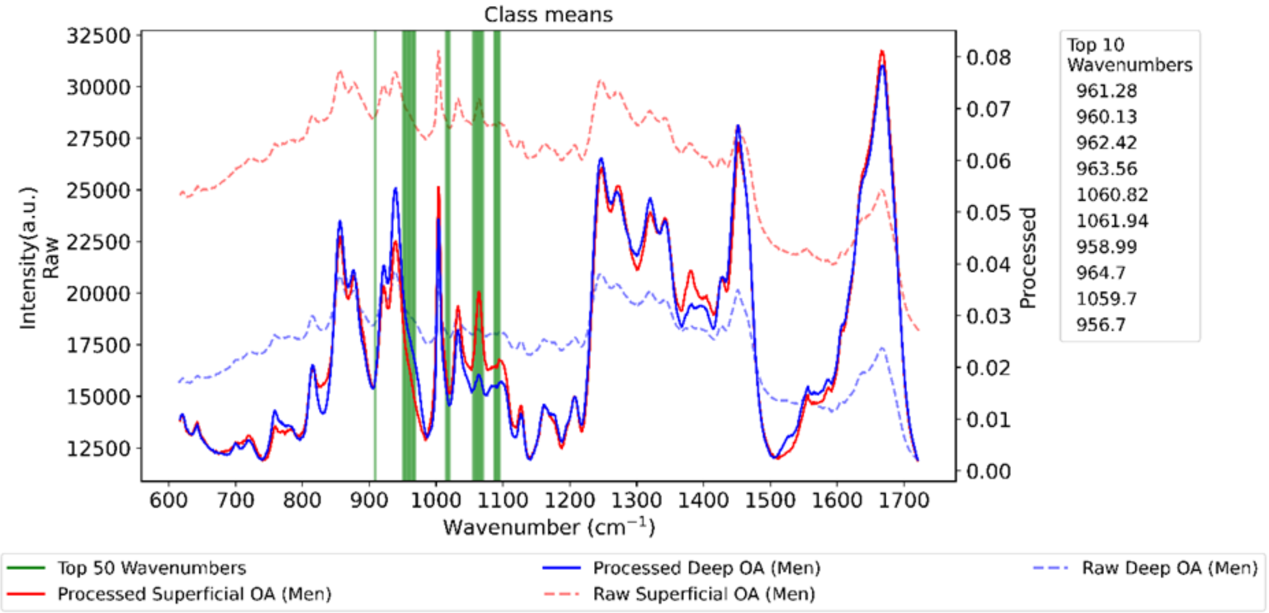

Figure B10 Top 50 wavenumbers highlighted by Multi-CNN at Region A: Superficial vs Deep OA (Men)

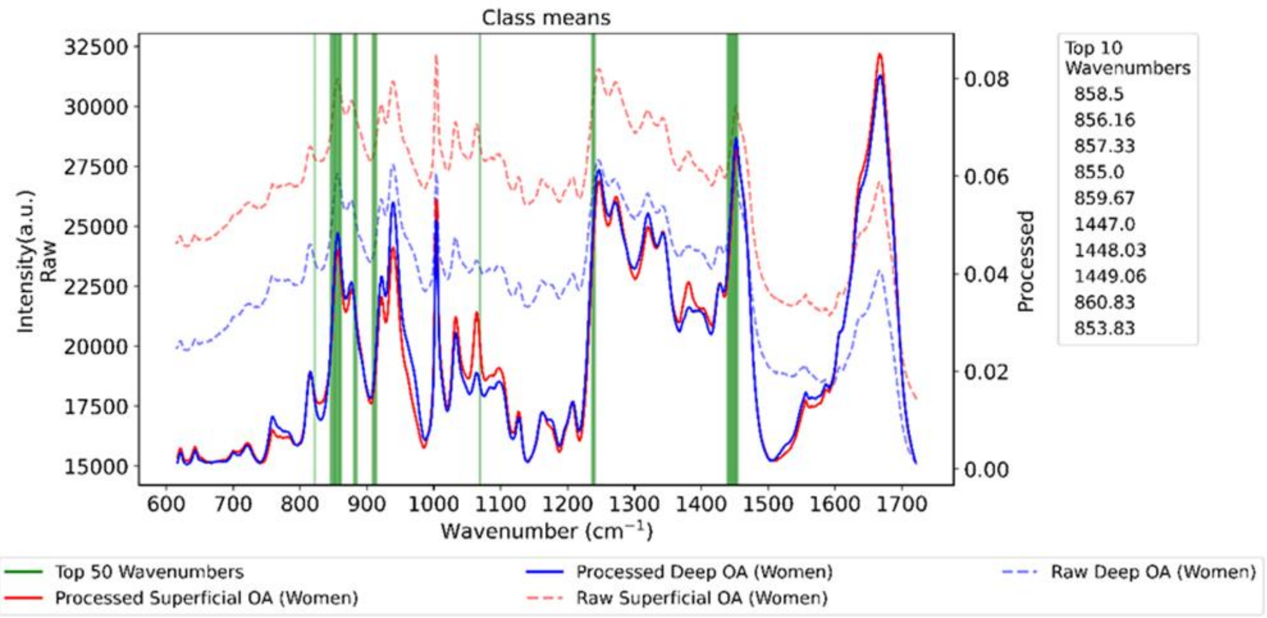

Figure B11 Top 50 wavenumbers highlighted by Multi-CNN at Region A: Superficial vs Deep OA (Women)

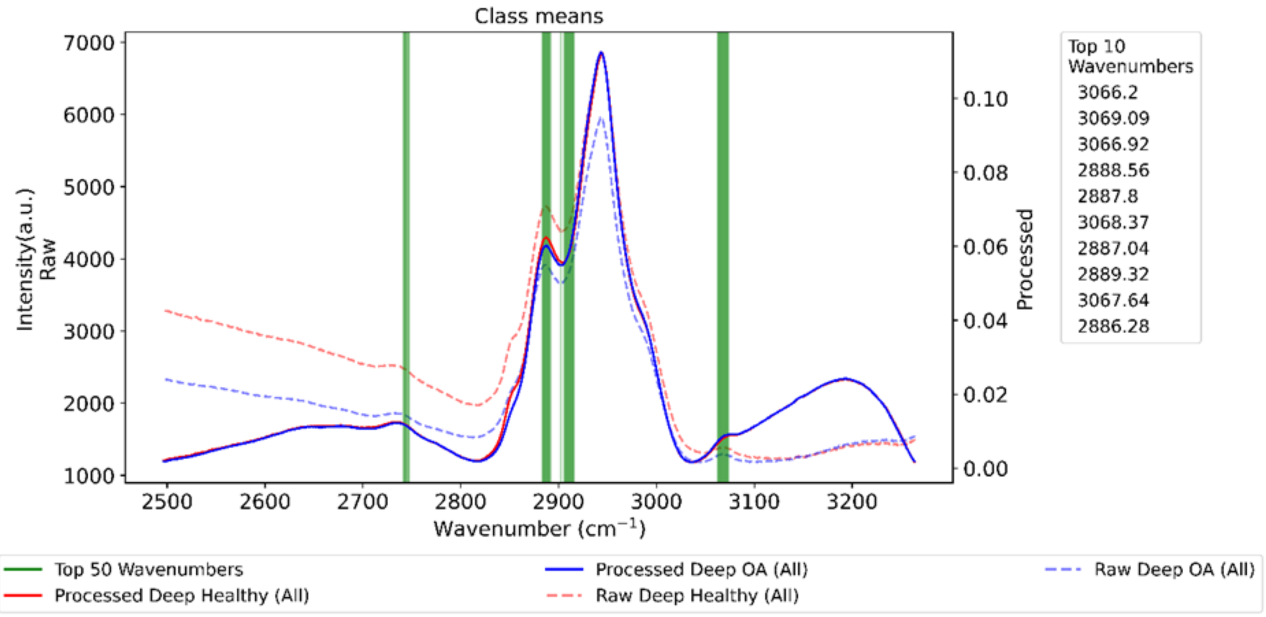

Figure B12 Top 50 wavenumbers highlighted by Multi-CNN at Region B: Deep Healthy vs OA (All)

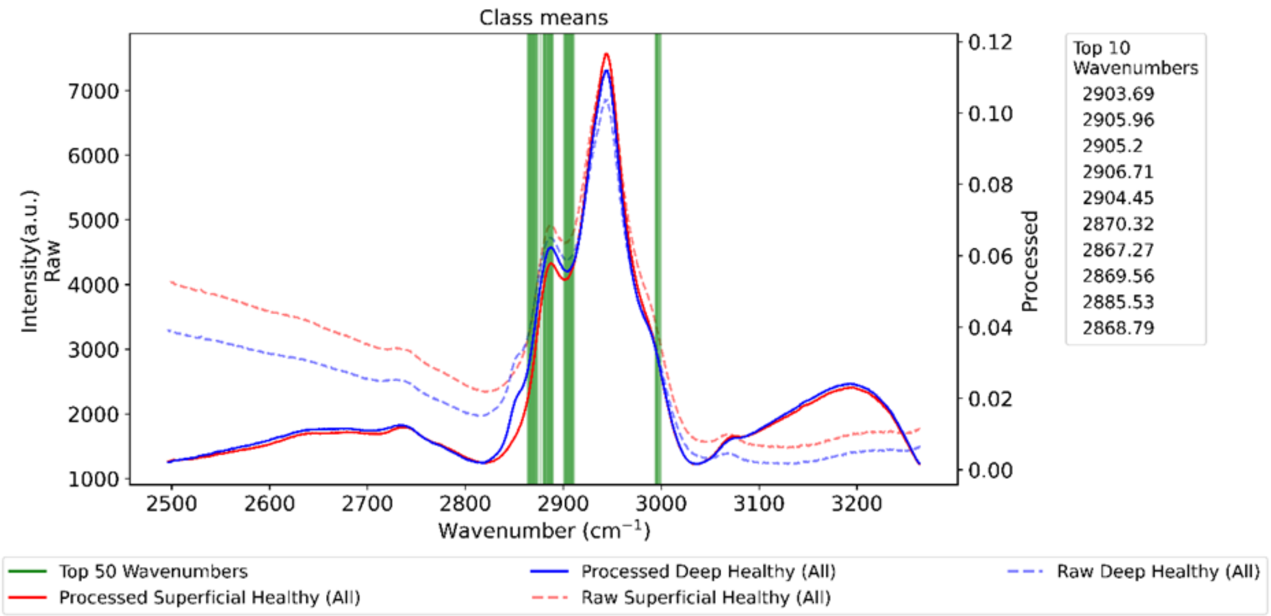

Figure B13 Top 50 wavenumbers highlighted by Multi-CNN at Region B: Superficial vs Deep Healthy (All)

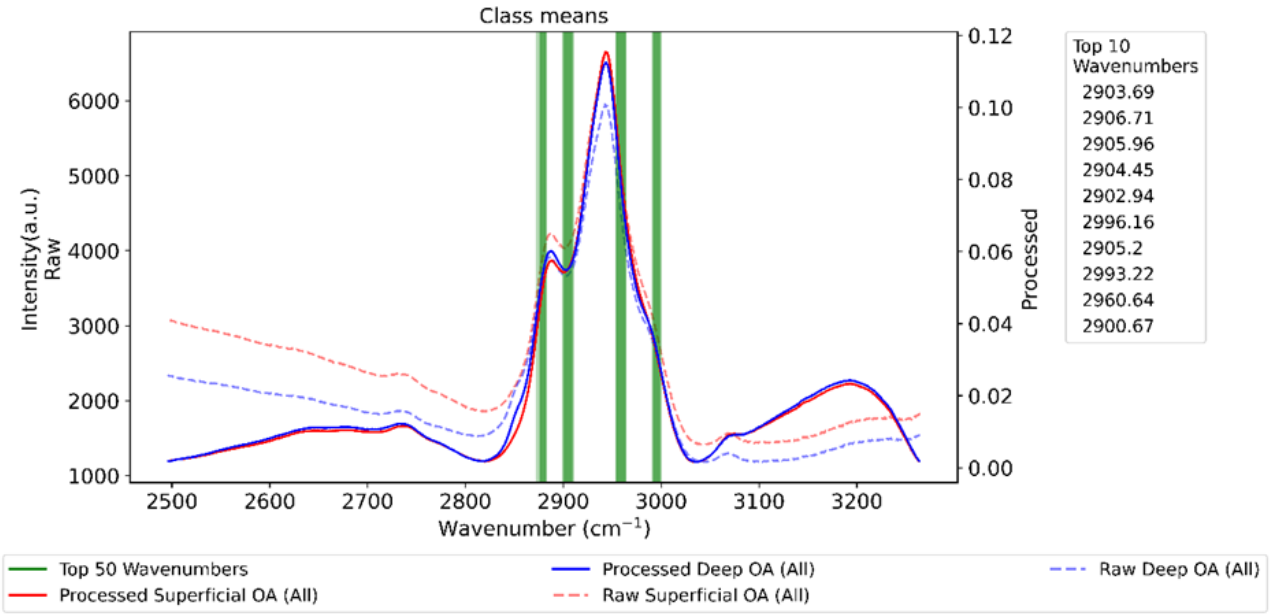

Figure B14 Top 50 wavenumbers highlighted by Multi-CNN at Region B: Superficial vs Deep OA (All)

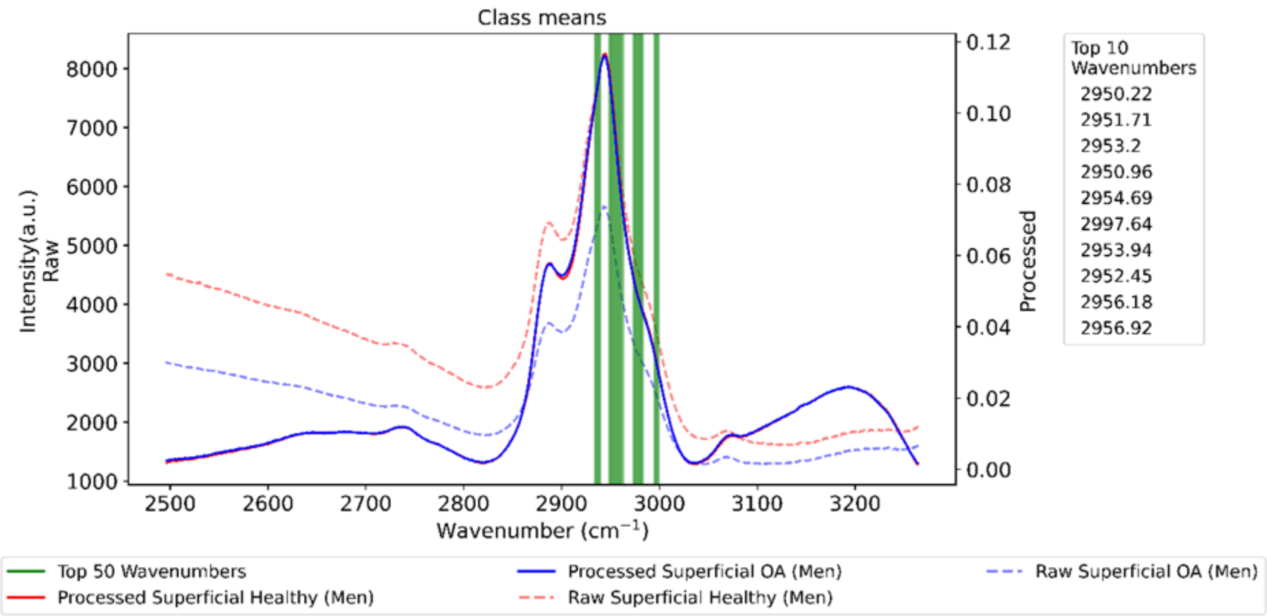

Figure B15 Top 50 wavenumbers highlighted by Multi-CNN at Region B: Superficial Healthy vs OA (Men)

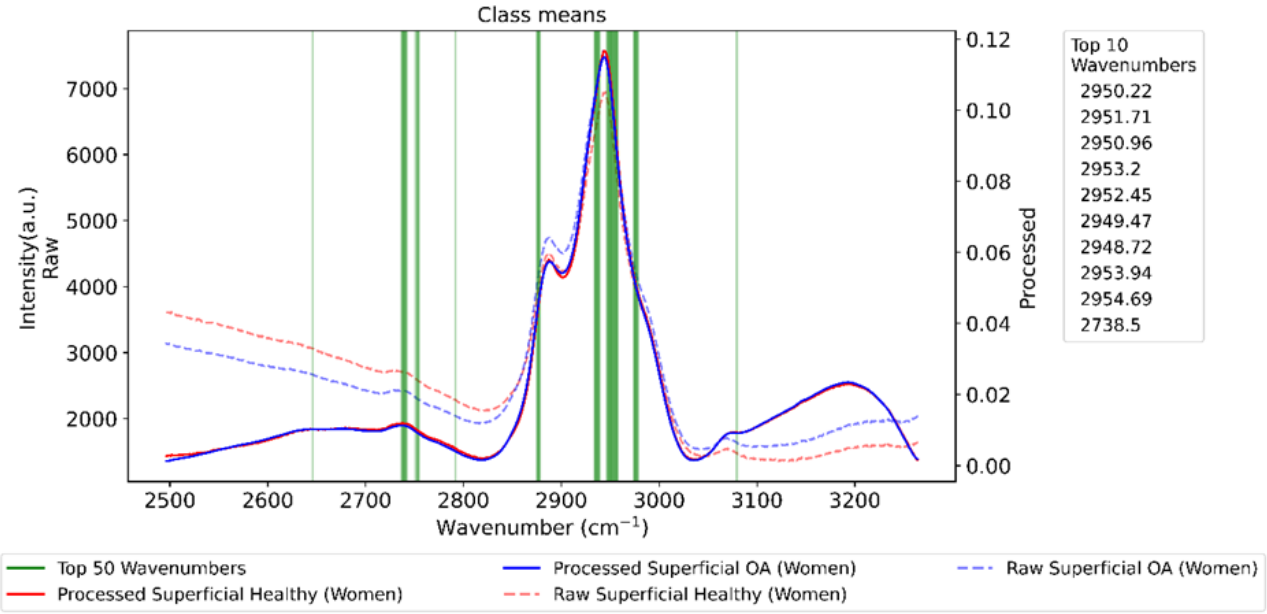

Figure B16 Top 50 wavenumbers highlighted by Multi-CNN at Region B: Superficial Healthy vs OA (Women)

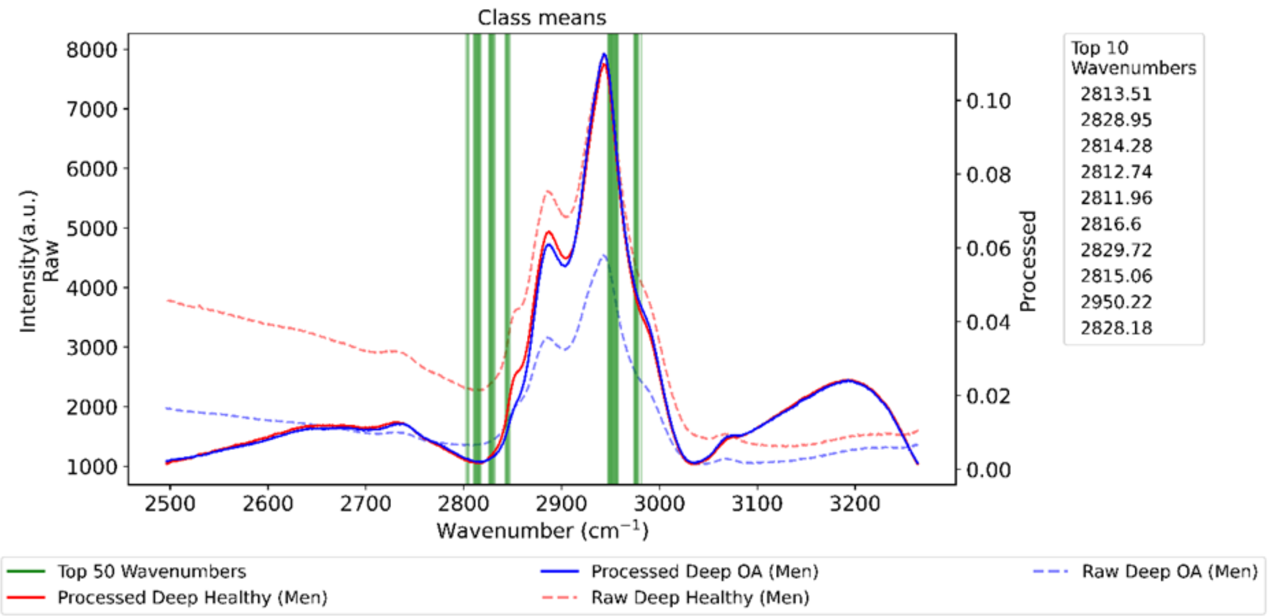

Figure B17 Top 50 wavenumbers highlighted by Multi-CNN at Region B: Deep Healthy vs OA (Men)

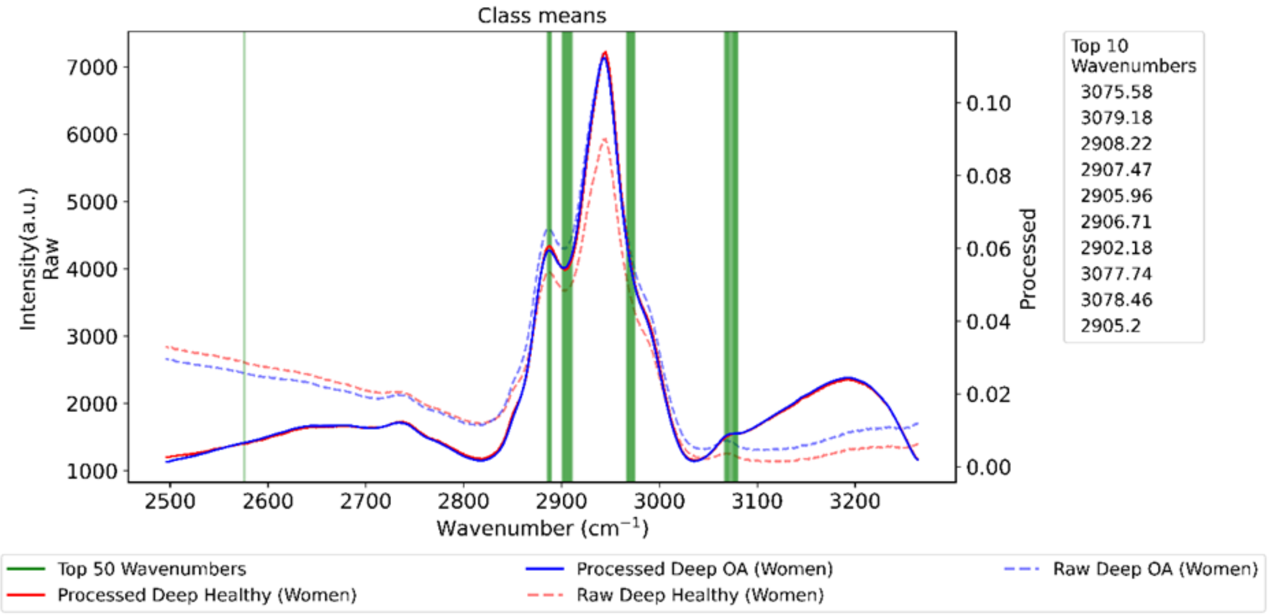

Figure B18 Top 50 wavenumbers highlighted by Multi-CNN at Region B: Deep Healthy vs OA (Women)

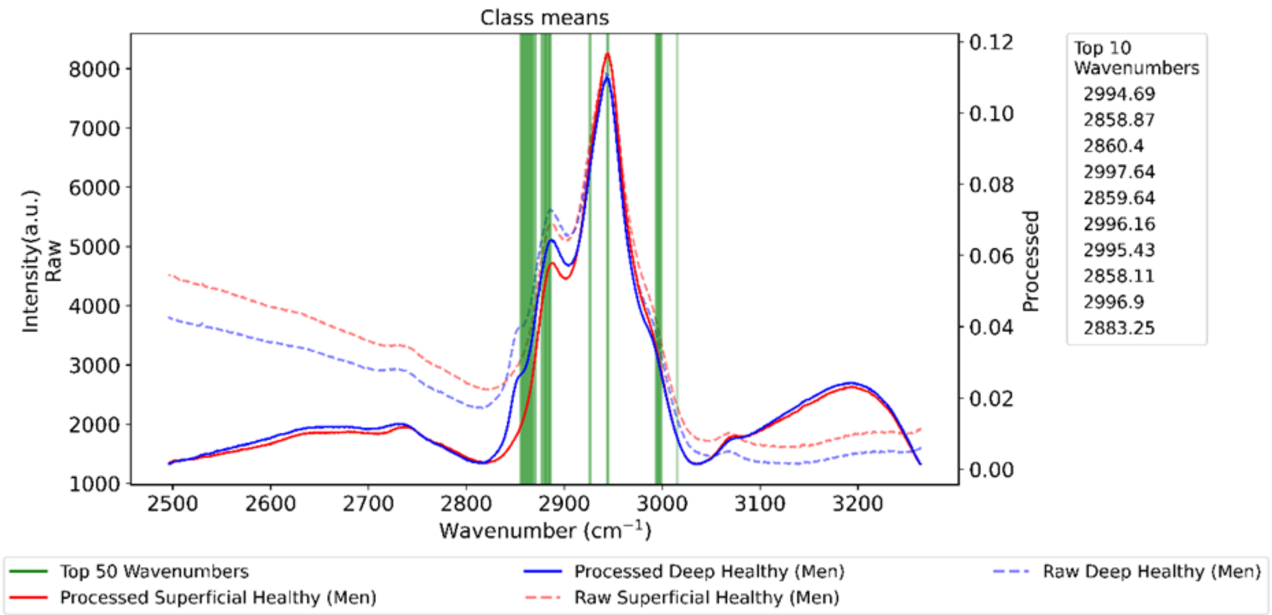

Figure B19 Top 50 wavenumbers highlighted by Multi-CNN at Region B: Superficial vs Deep Healthy (Men)

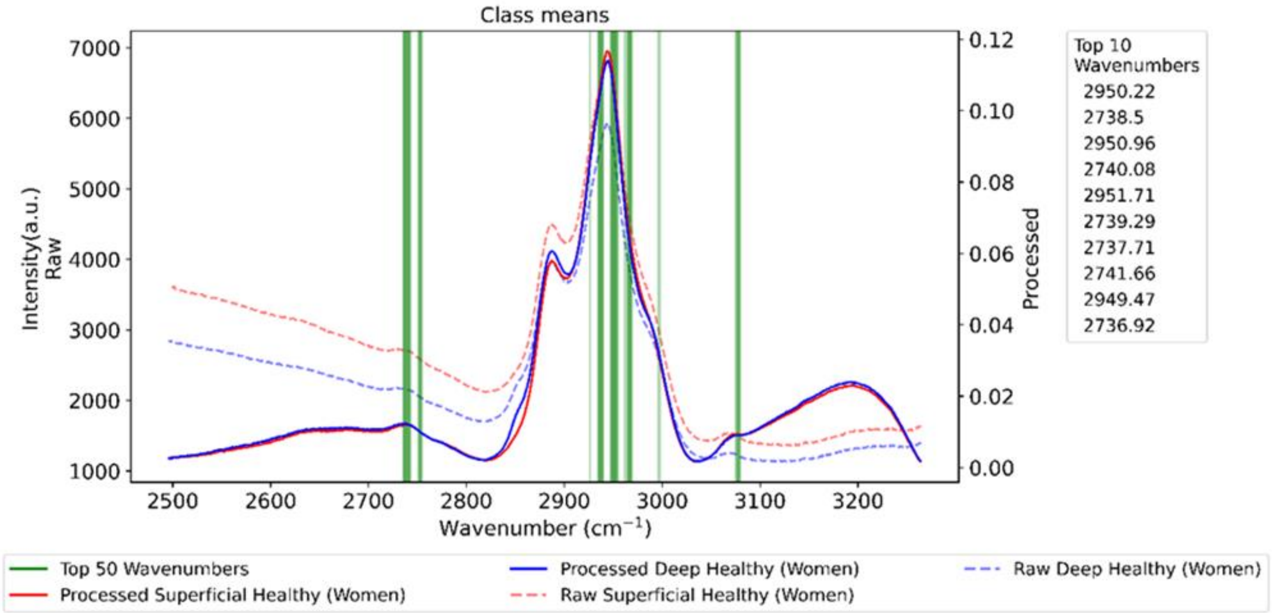

Figure B20 Top 50 wavenumbers highlighted by Multi-CNN at Region B: Superficial vs Deep Healthy (Women)

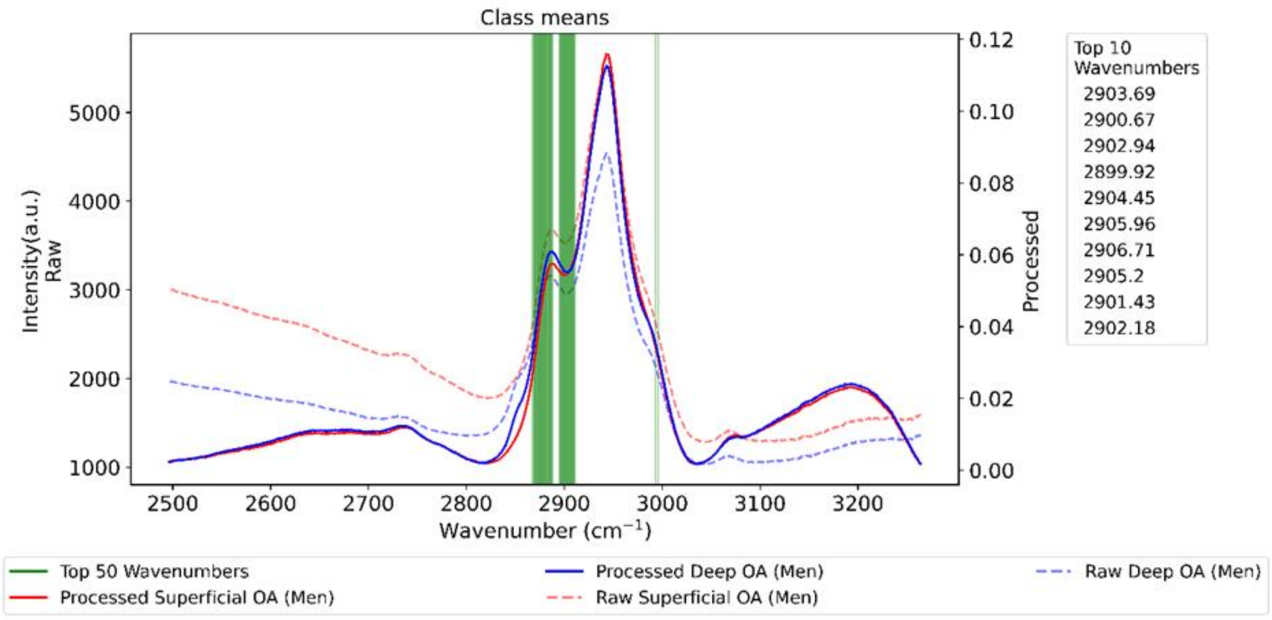

Figure B21 Top 50 wavenumbers highlighted by Multi-CNN at Region B: Superficial vs Deep OA (Men)

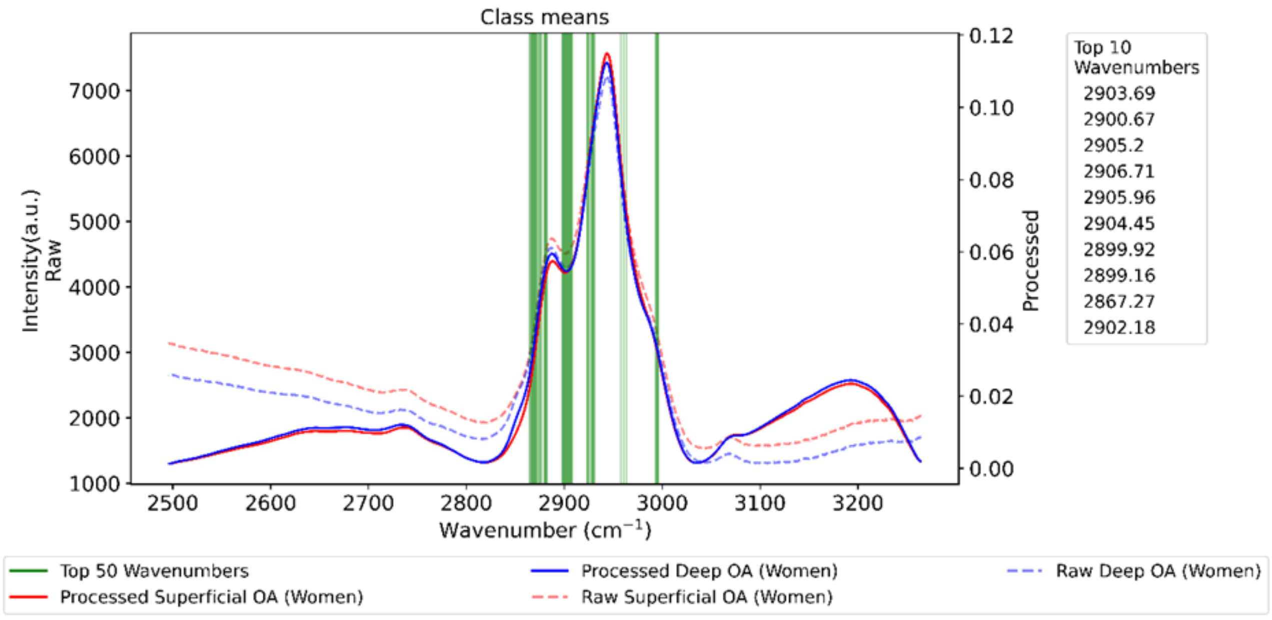

Figure B22 Top 50 wavenumbers highlighted by Multi-CNN at Region B: Superficial vs Deep OA (Women)

Appendix C

Evaluation of Machine Learning methodologies on selected number of features

Features are reranked and later selected according to their importance highlighted by our network. Results shown are the average score from the 6-fold cross validation.

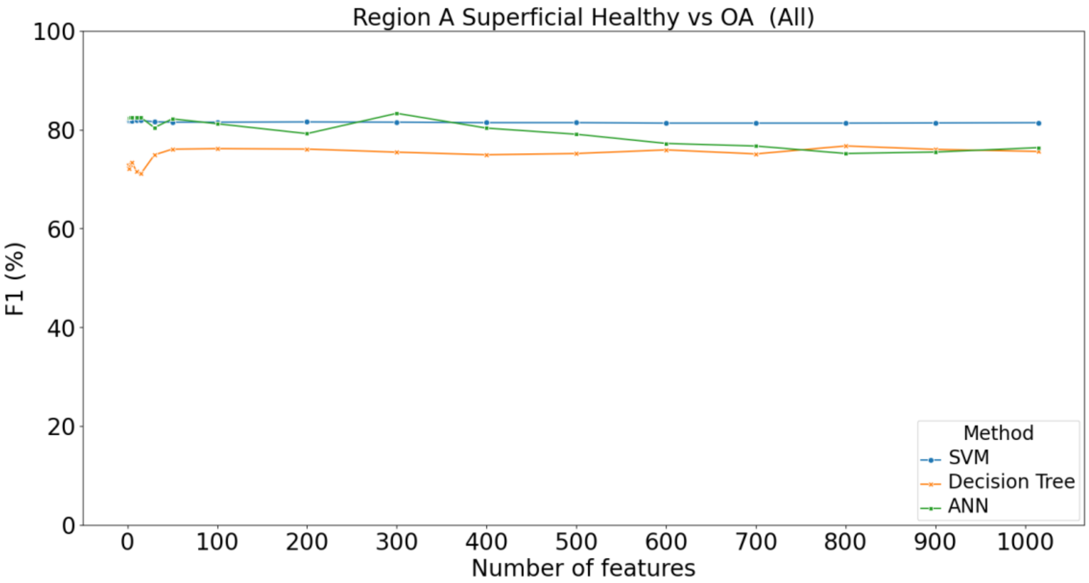

Figure C1 Feature selection using SVM, Decision Tree or ANN at Region A: Superficial Healthy vs OA (All)

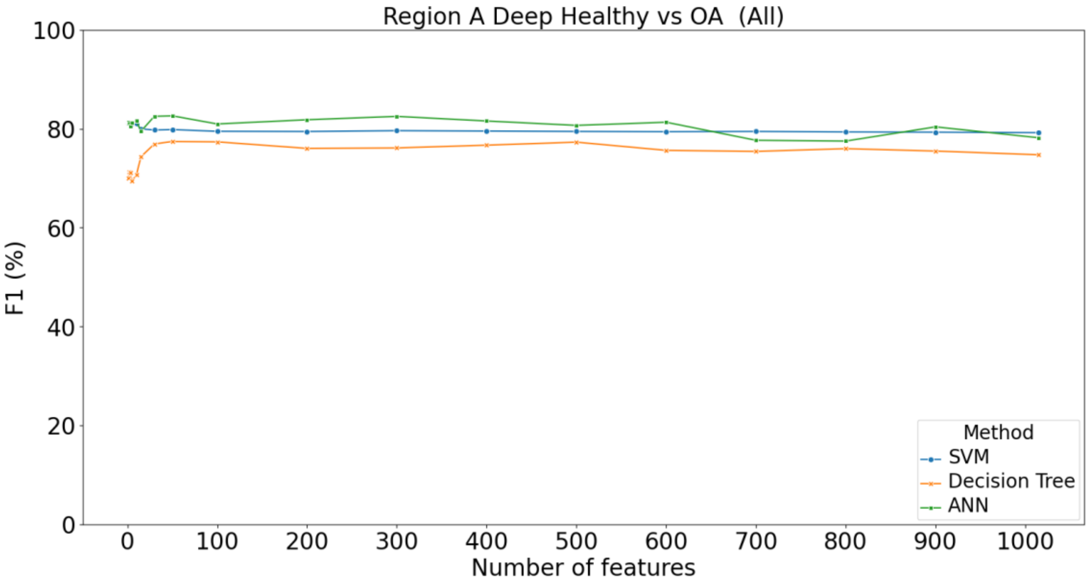

Figure C2 Feature selection using SVM, Decision Tree or ANN at Region A: Deep Healthy vs OA (All)

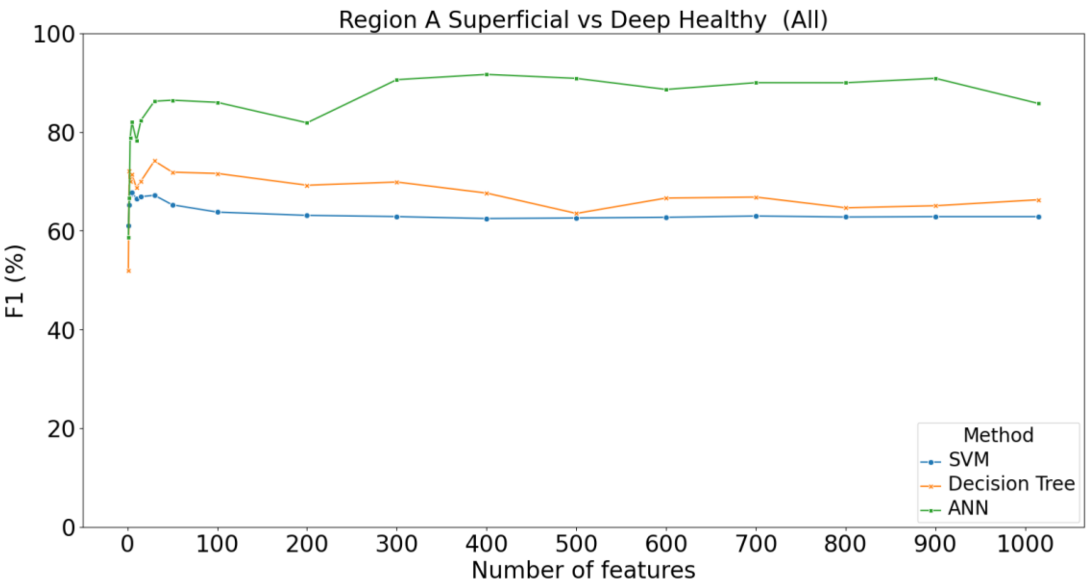

Figure C3 Feature selection using SVM, Decision Tree or ANN at Region A: Superficial vs Deep Healthy (All)

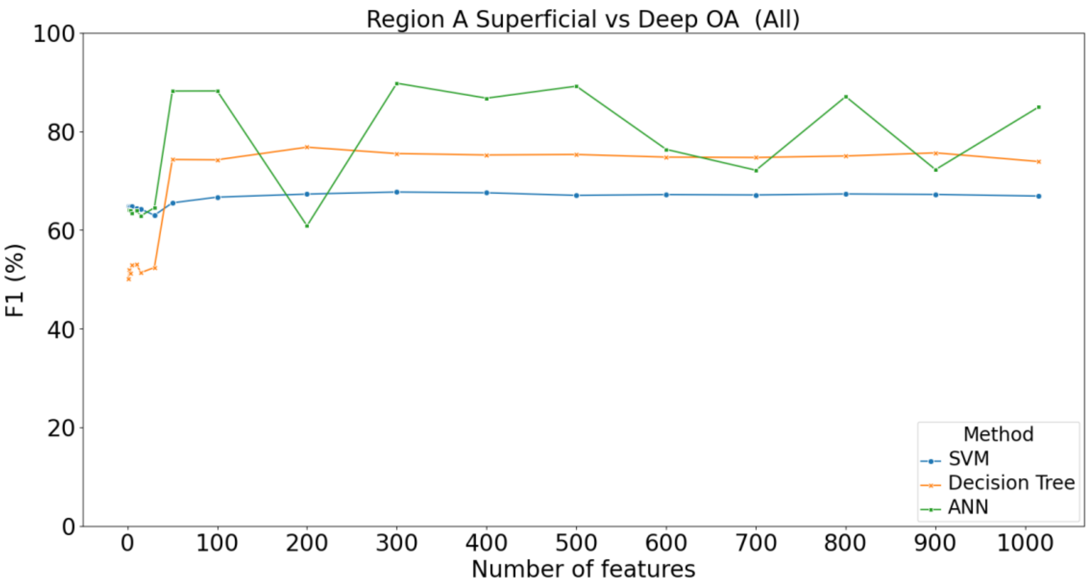

Figure C4 Feature selection using SVM, Decision Tree or ANN at Region A: Superficial vs Deep OA (All)

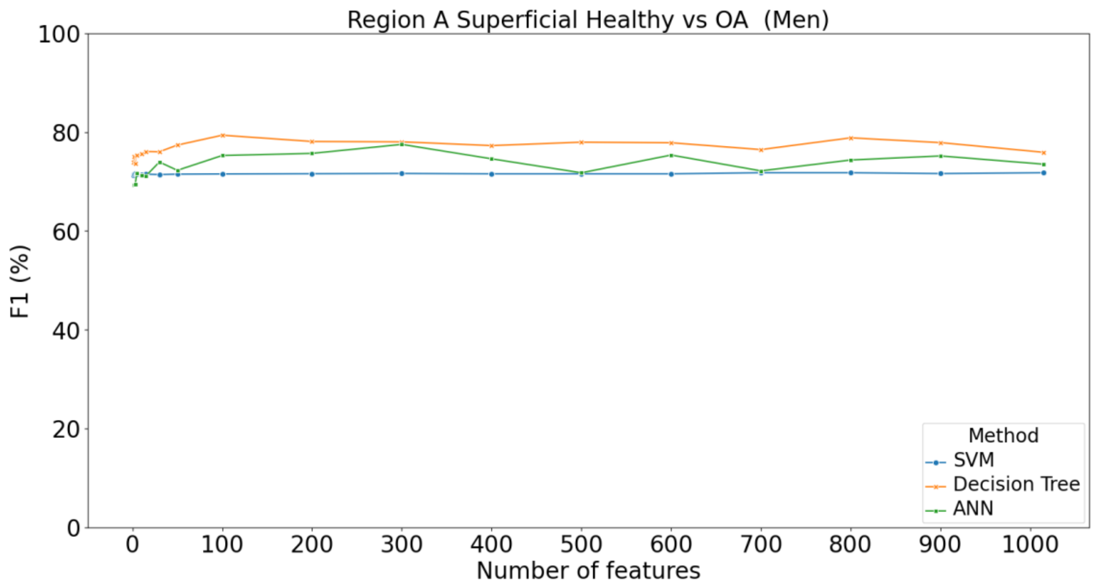

Figure C5 Feature selection using SVM, Decision Tree or ANN at Region A: Superficial Healthy vs OA (Men)

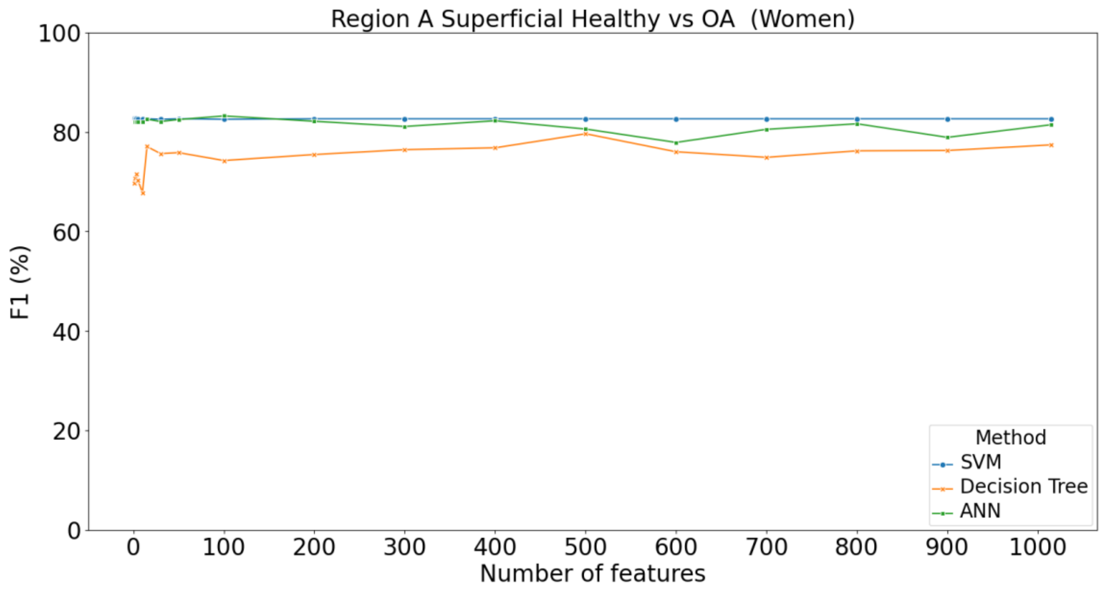

Figure C6 Feature selection using SVM, Decision Tree or ANN at Region A: Superficial Healthy vs OA (Women)

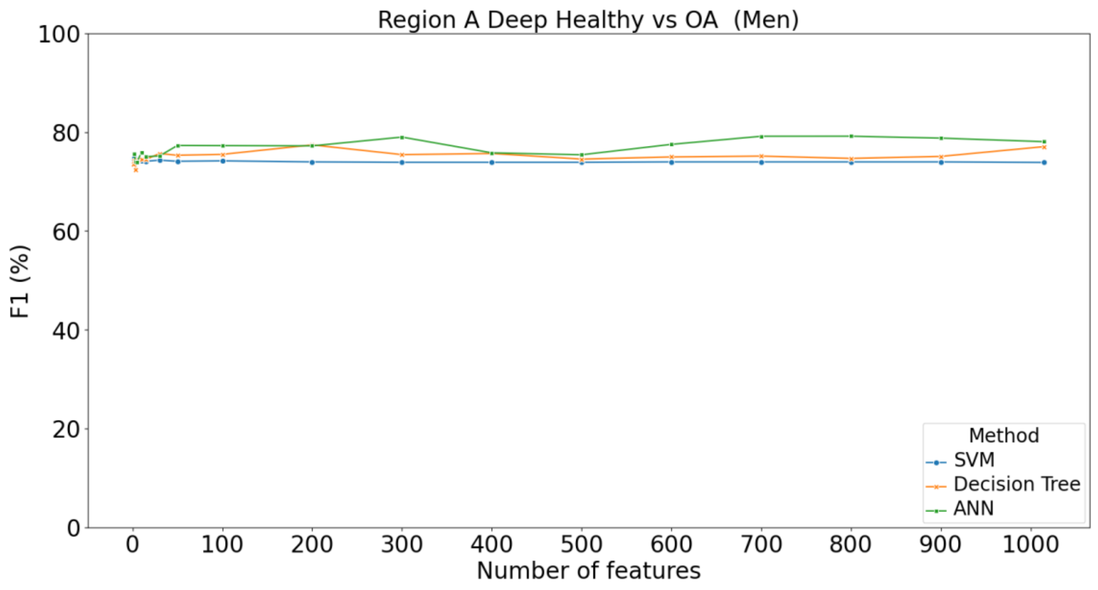

Figure C7 Feature selection using SVM, Decision Tree or ANN at Region A: Deep Healthy vs OA (Men)

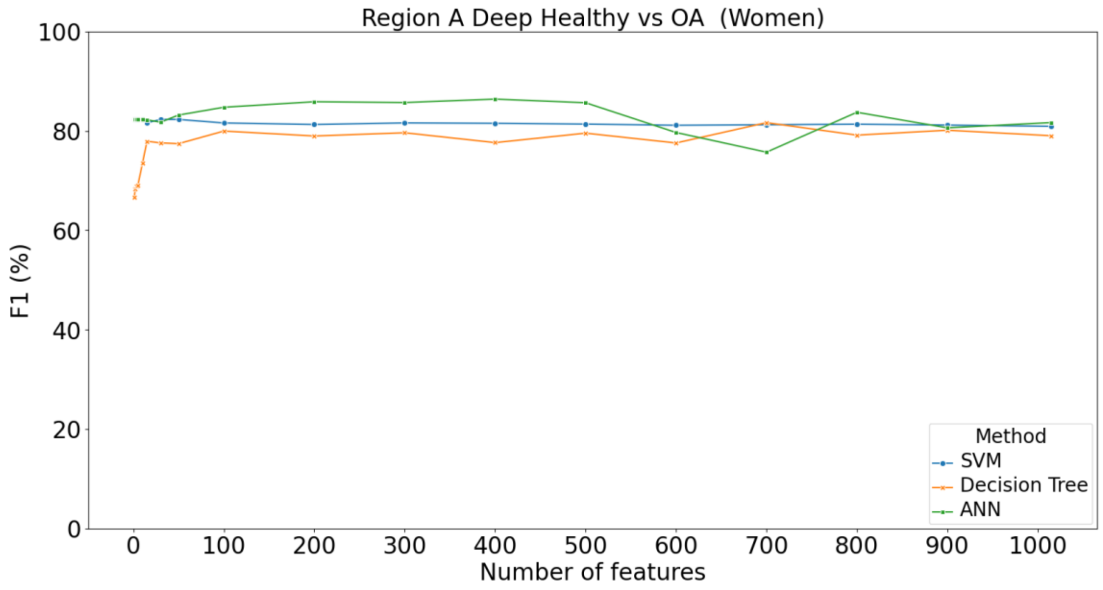

Figure C8 Feature selection using SVM, Decision Tree or ANN at Region A: Deep Healthy vs OA (Women)

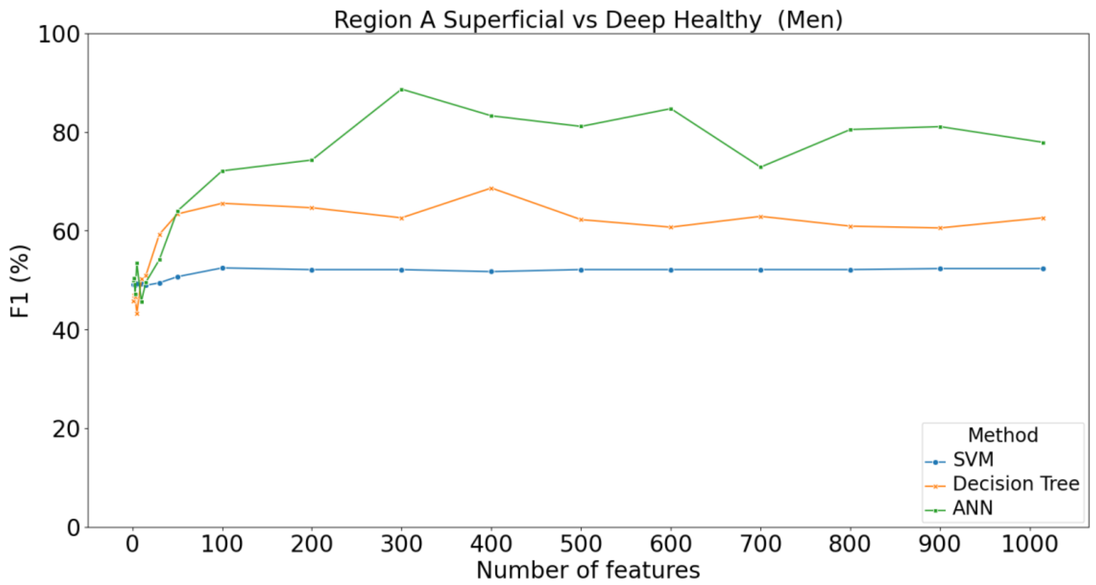

Figure C9 Feature selection using SVM, Decision Tree or ANN at Region A: Superficial vs Deep Healthy (Men)

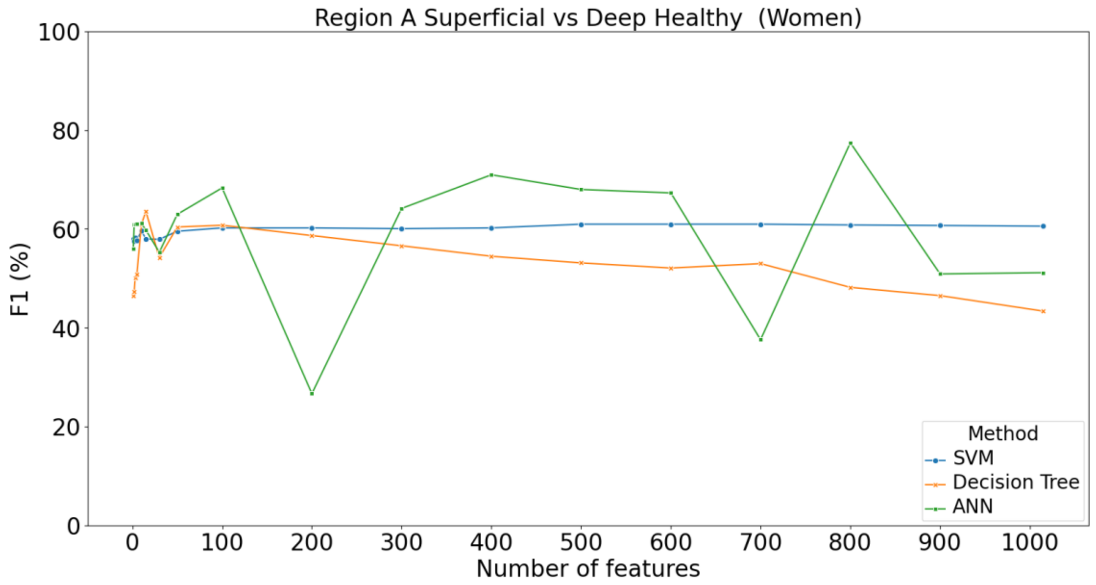

Figure C10 Feature selection using SVM, Decision Tree or ANN at Region A: Superficial vs Deep Healthy (Women)

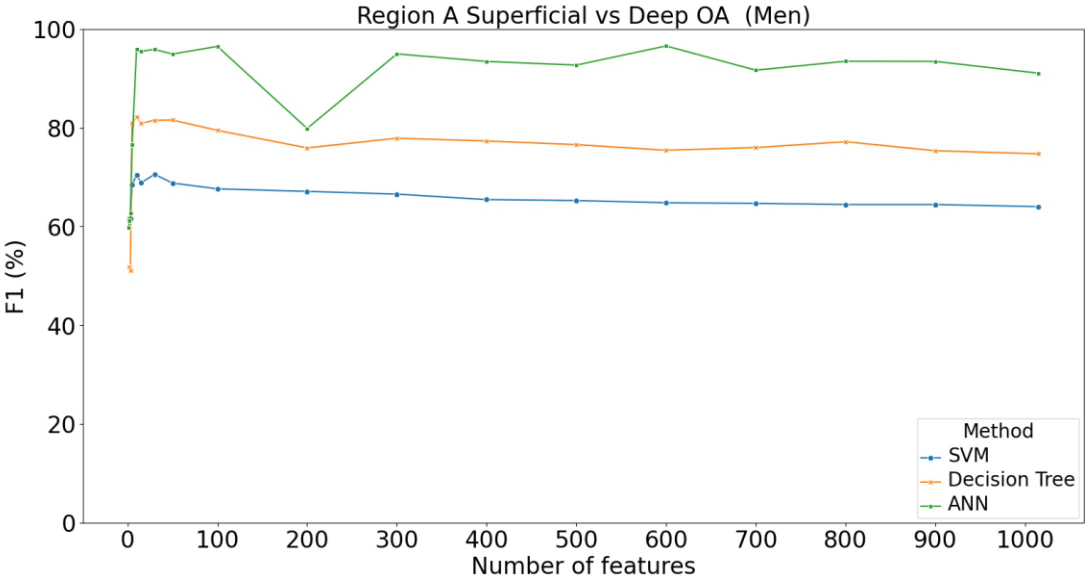

Figure C11 Feature selection using SVM, Decision Tree or ANN at Region A: Superficial vs Deep OA (Men)

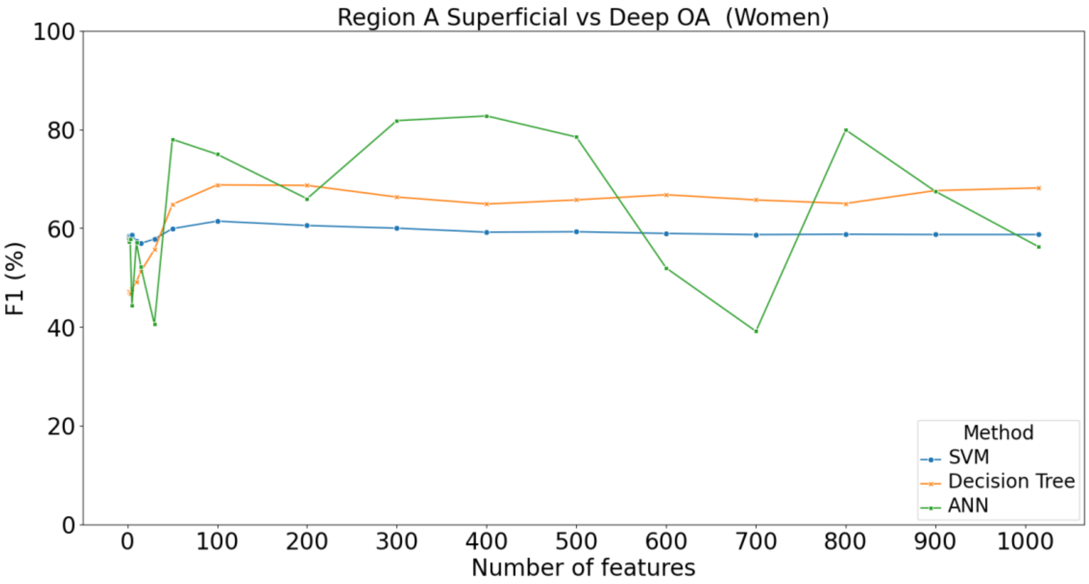

Figure C12 Feature selection using SVM, Decision Tree or ANN at Region A: Superficial vs Deep OA (Women)

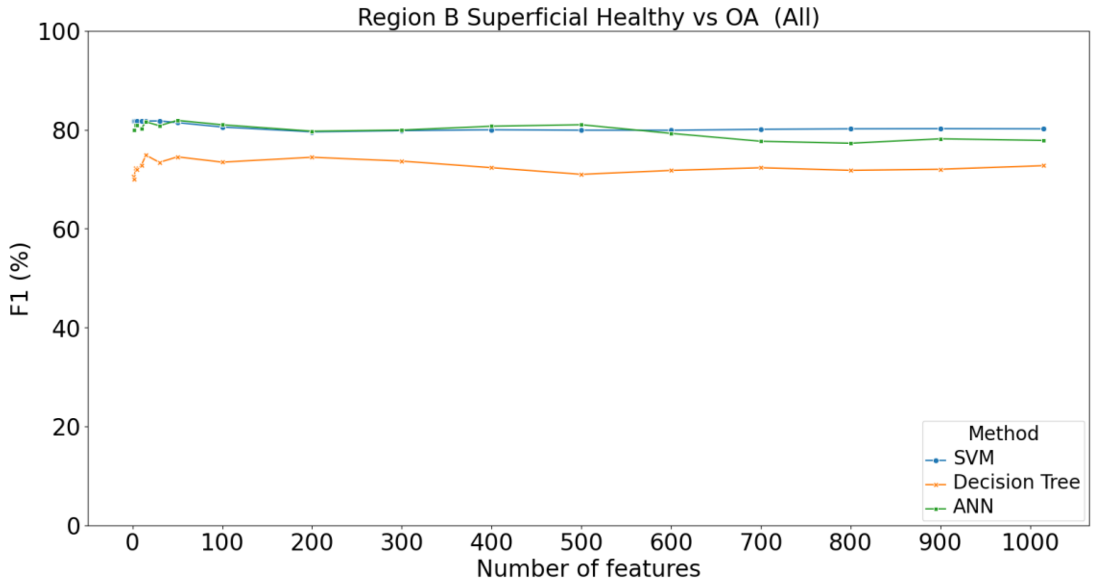

Figure C13 Feature selection using SVM, Decision Tree or ANN at Region B: Superficial Healthy vs OA (All)

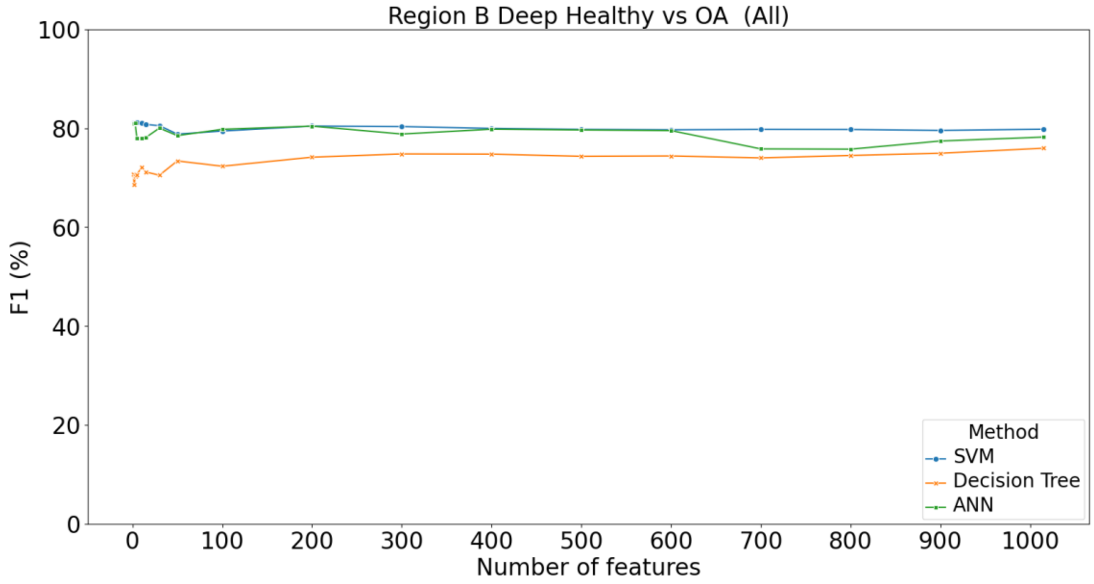

Figure C14 Feature selection using SVM, Decision Tree or ANN at Region B: Deep Healthy vs OA (All)

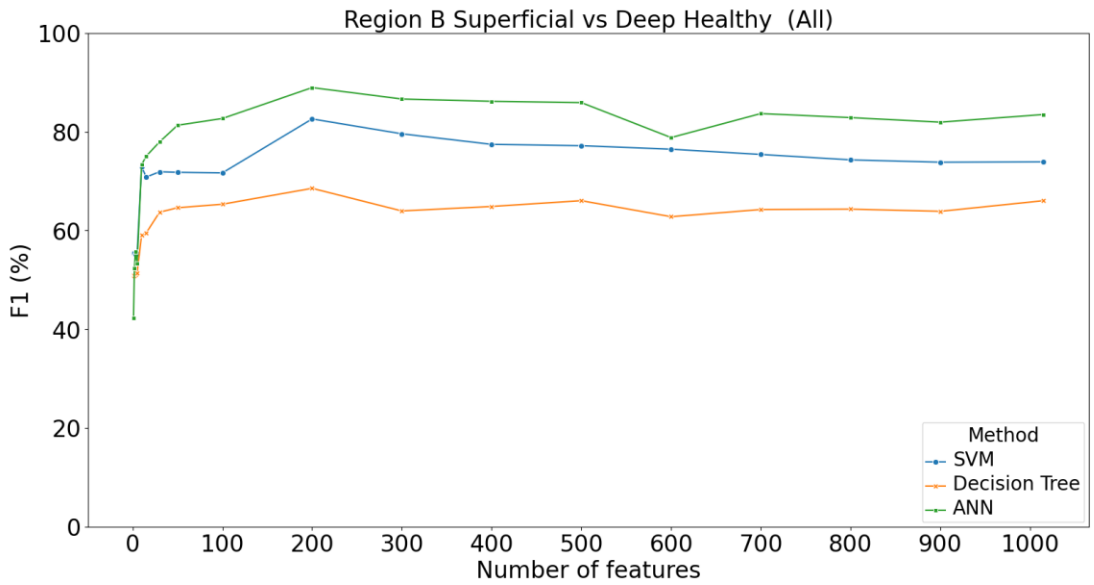

Figure C15 Feature selection using SVM, Decision Tree or ANN at Region B: Superficial vs Deep Healthy (All)

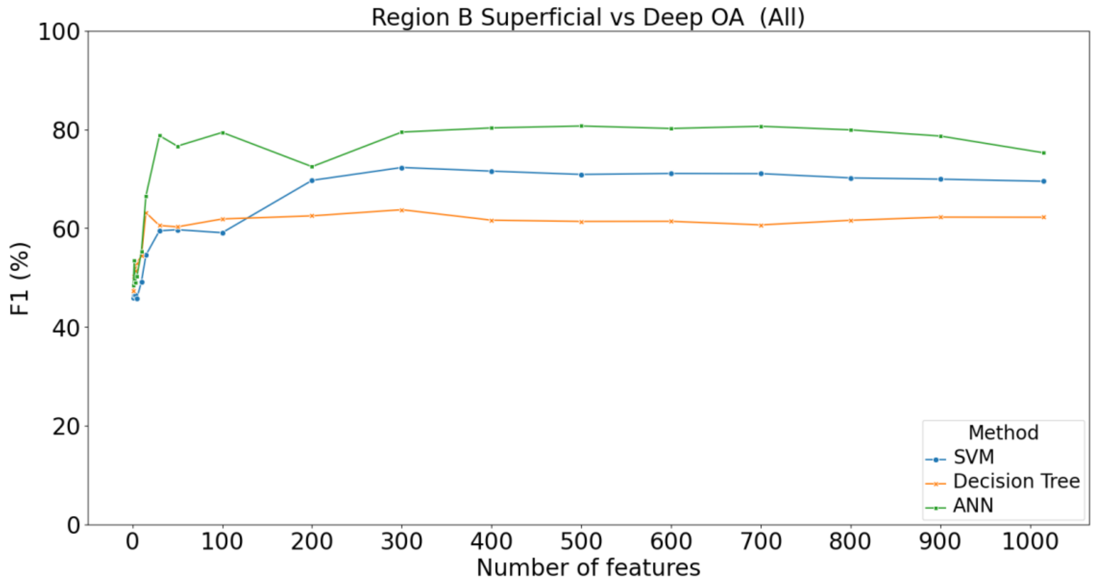

Figure C16 Feature selection using SVM, Decision Tree or ANN at Region B: Superficial vs Deep OA (All)

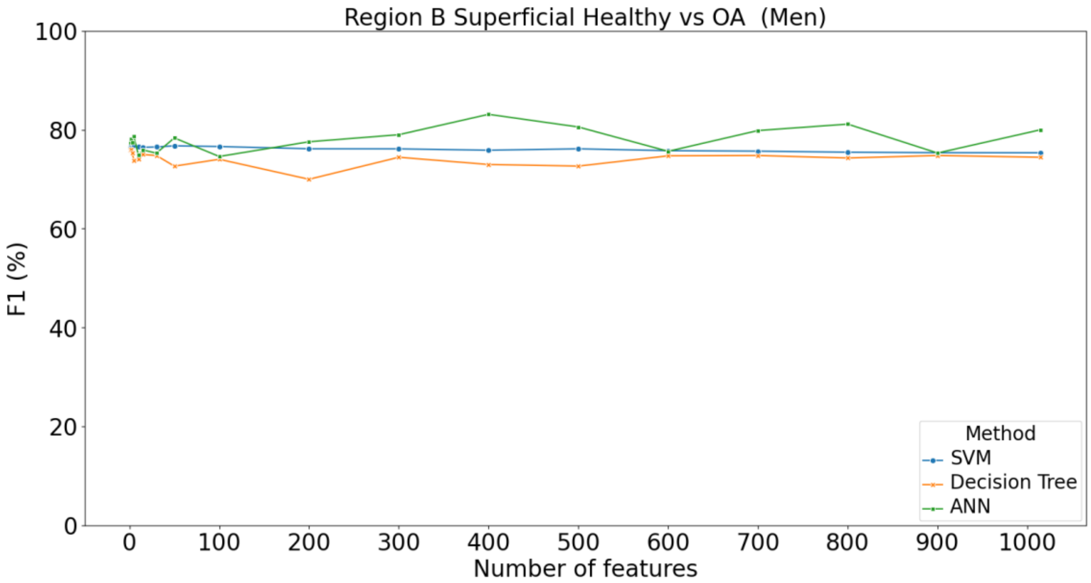

Figure C17 Feature selection using SVM, Decision Tree or ANN at Region B: Superficial Healthy vs OA (Men)

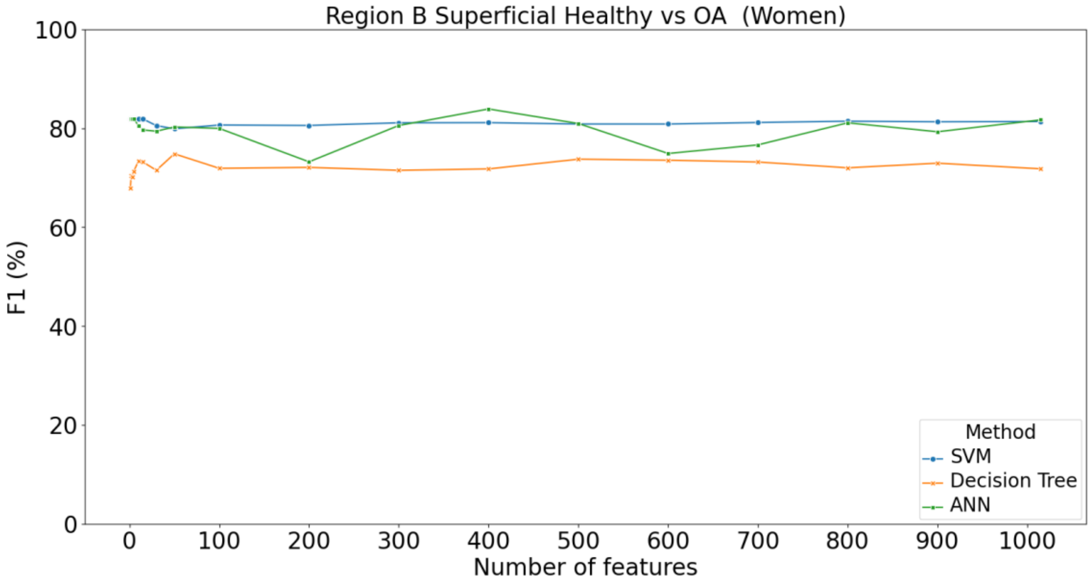

Figure C18 Feature selection using SVM, Decision Tree or ANN at Region B: Superficial Healthy vs OA (Women)

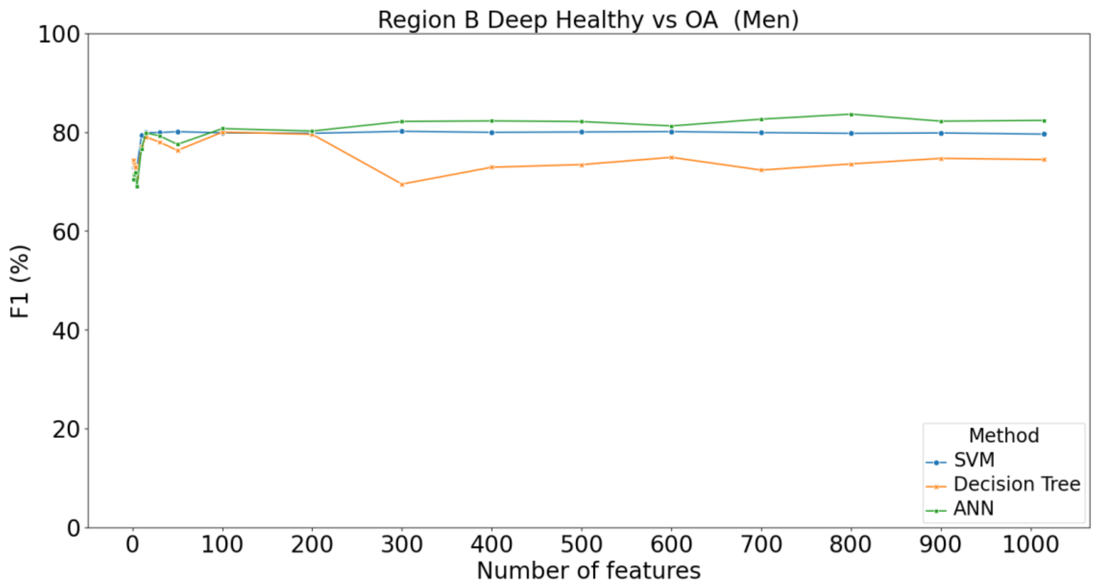

Figure C19 Feature selection using SVM, Decision Tree or ANN at Region B: Deep Healthy vs OA (Men)

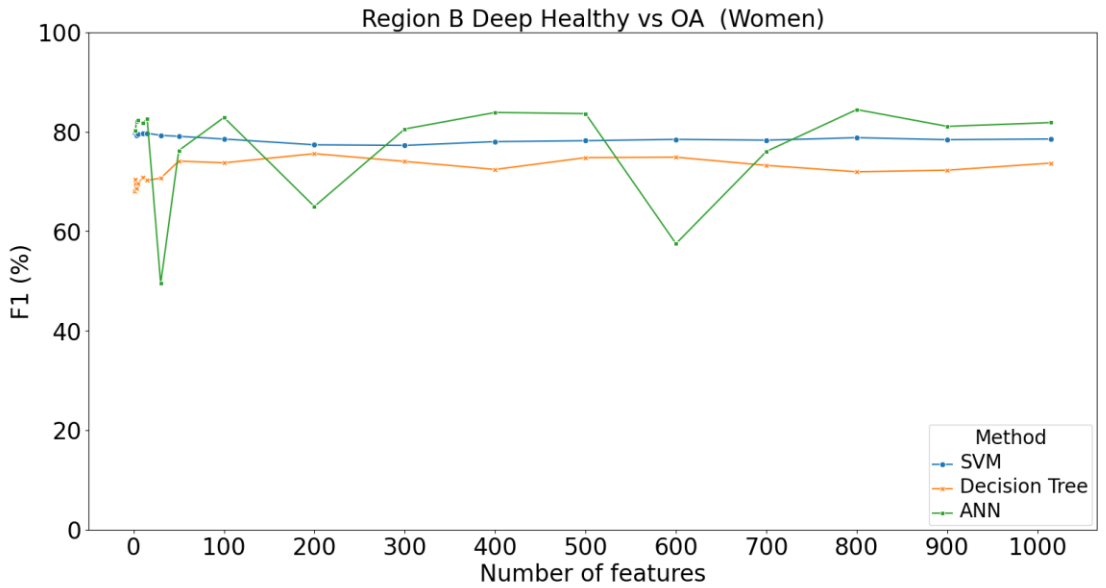

Figure C20 Feature selection using SVM, Decision Tree or ANN at Region B: Deep Healthy vs OA (Women)

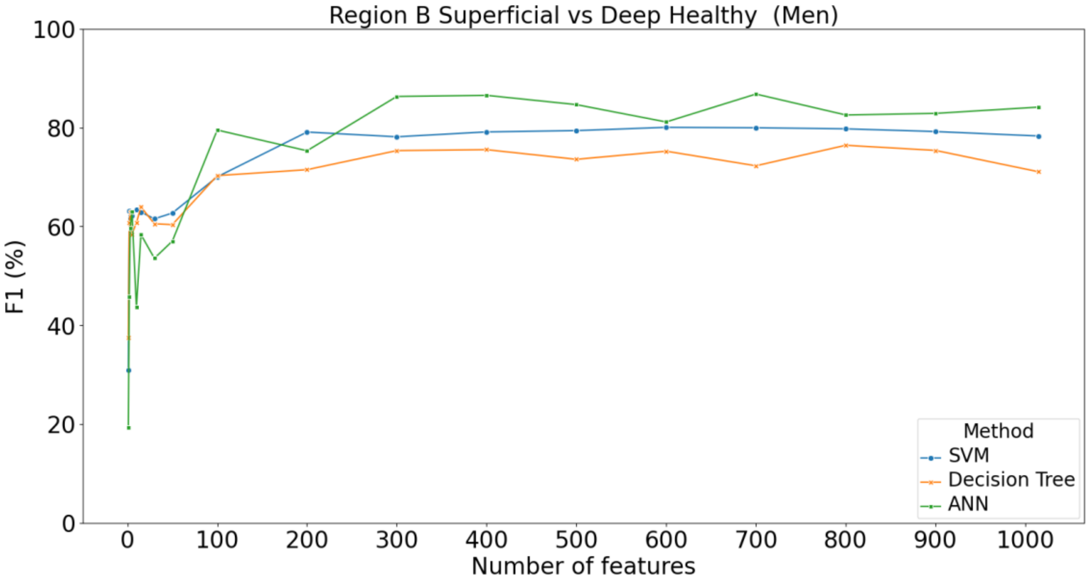

Figure C21 Feature selection using SVM, Decision Tree or ANN at Region B: Superficial vs Deep Healthy (Men)

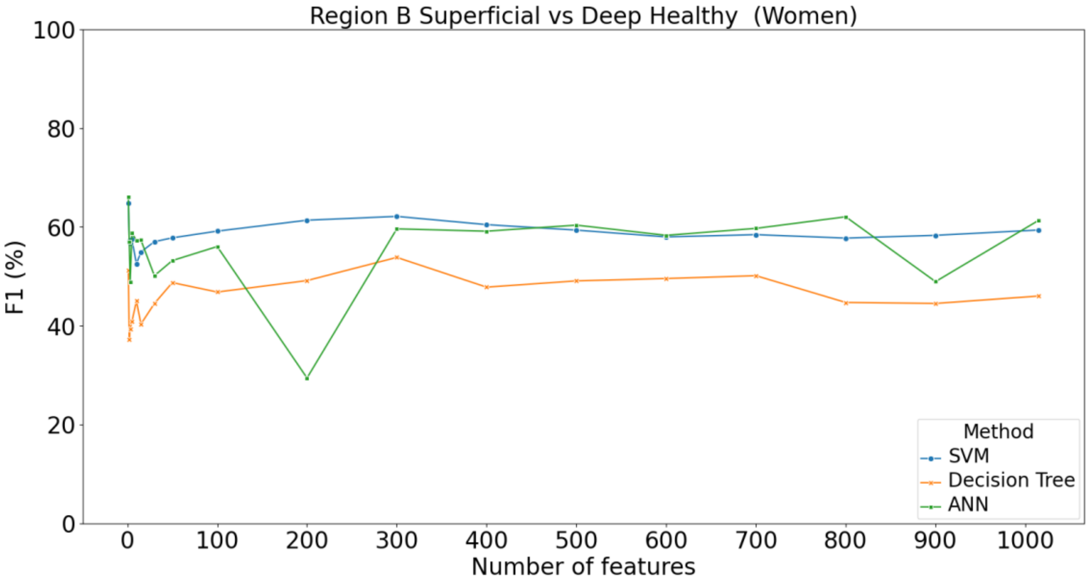

Figure C22 Feature selection using SVM, Decision Tree or ANN at Region B: Superficial vs Deep Healthy (Women)

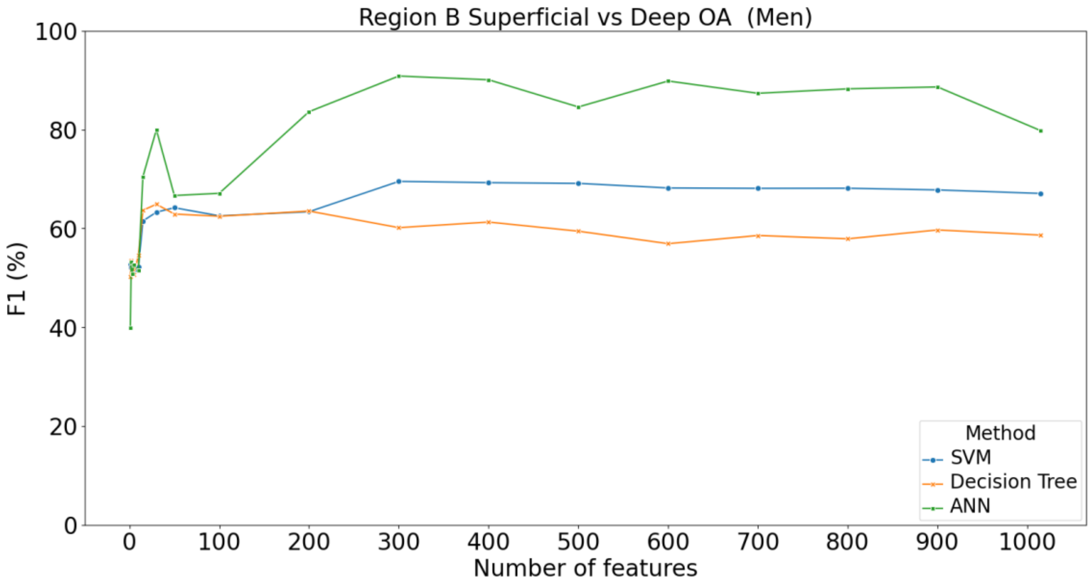

Figure C23 Feature selection using SVM, Decision Tree or ANN at Region B: Superficial vs Deep OA (Men)

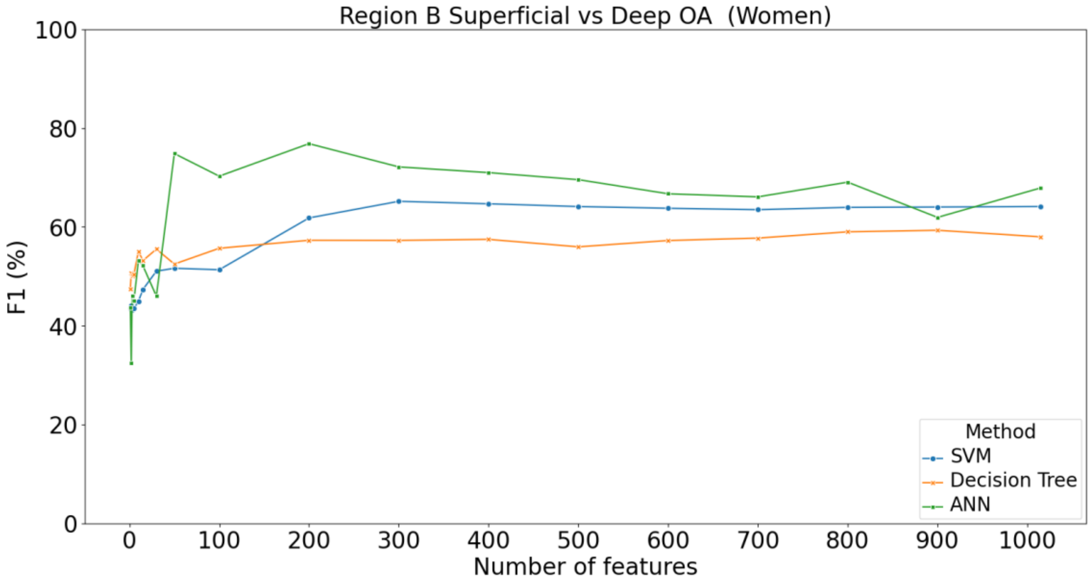

Figure C24 Feature selection using SVM, Decision Tree or ANN at Region B: Superficial vs Deep OA (Women)
